# TST Score Helper: An Open-Source Graphical User Interface for Assisted Manual Scoring of the Tail Suspension Test

**DOI:** 10.1523/ENEURO.0318-25.2026

**Published:** 2026-04-08

**Authors:** Sydney E. Triplett, Chenxing Li, Paul Sanz, Shasha Bai, Levi B. Wood, Erin M. Buckley

**Affiliations:** ^1^The Wallace H. Coulter Department of Biomedical Engineering, Georgia Institute of Technology/Emory University, Atlanta, Georgia 30318; ^2^Departments of Chemistry, Emory University, Atlanta, Georgia 30322; ^3^Pediatrics, Emory University, Atlanta, Georgia 30322; ^4^George W. Woodruff School of Mechanical Engineering, Georgia Institute of Technology, Atlanta, Georgia 30318; ^5^Parker H. Petit Institute for Bioengineering and Bioscience, Georgia Institute of Technology, Atlanta, Georgia 30318; ^6^Children’s Healthcare of Atlanta, Children’s Research Scholar, Atlanta, Georgia 30329

**Keywords:** behavioral testing, GUI, MATLAB GUI, mouse, tail suspension test

## Abstract

The tail suspension test (TST) is a well-known rodent behavioral test that assesses stress and depressive-like behavior. While several automatic tail suspension test scoring programs have emerged, many researchers still prefer a manual scoring method for accuracy and reliability. However, manual scoring can introduce significant errors. Thus, in this work, we present a novel graphical user interface that assists in the manual scoring process to minimize possibility for errors. The GUI, which we refer to as “TST Score Helper,” minimizes errors through consolidation of the TST scoring procedure into a single cohesive program. Further, a rescore mode enhances rigor by enabling comparison of two different scorers’ mobility status timelines and rereview of periods of disagreement. In a cohort of 64 male and 45 female mice subject to closed head injury or sham injury, we demonstrate the challenges with manual scoring and we characterize performance of the TST Score Helper program. The results show how this program can reduce sources of manual scoring error and improve the fidelity of results.

## Significance Statement

The tail suspension test (TST) is widely used to study depressive-like behavior in rodents, but scoring is a manual process that is slow, nuanced, and prone to error. We developed TST Score Helper, a simple software tool that makes scoring more reliable. Its key feature is a rescore mode that combines two raters’ scores for the same trial, flags disagreements, and guides raters to reach a final score. By simplifying the process and improving consistency, TST Score Helper enhances the rigor and reproducibility of the TST.

## Introduction

The tail suspension test (TST) is a behavioral assessment widely used in rodents to measure the effect of antidepressant interventions (Steru et al., 1985; O’Leary and Cryan, 2009; Belovicova et al., 2017). TST is also commonly used as an outcome marker for antidepressive-like behavior following experimental manipulation. In TST, the subject is suspended by the tail for 6 min, during which time the subject will initially struggle to escape. Over time, escape attempts and related behaviors subside, and episodes of immobility emerge (Lad et al., 2007; Can et al., 2012). Immobility duration is scored by subtracting the total time the subject spends mobile from the 6 min total duration. Immobility duration has been shown to be increased in animals after experimental interventions like sleep deprivation, and it has been found to be reduced after treatment with antidepressants (Vaugeois et al., 1996; El Yacoubi et al., 2003; Cryan et al., 2005; Li et al., 2024). Given the relative ease of implementing the test and the simplicity of scoring, TST is commonly used as a rapid screening tool to assess the influence of experimental and pharmacological interventions on depressive-like behavior (Cryan et al., 2005).

Unfortunately, scoring TST is prone to several sources of errors. First, automated scoring is impeded by complicated behavior categorization that includes consideration of the subject's whole body versus limb movements, intentionality in movement, and consistent movement categorization. Some behaviors that involve mobility are categorized as immobility on TST, e.g., oscillatory swinging due to momentum resulting from past movements or movements confined to two feet. Thus, scoring must be performed by trained personnel. However, human scoring introduces another source of unavoidable error. Observer bias in mobility scoring combined with long trial length that requires sharp attention to detail for an extended period of time can cause errors in analysis. While promising automated scoring methods combatting these issues have emerged (Juszczak et al., 2006; Bohnslav et al., 2021; Nandi et al., 2021; Isik and Unal, 2023; Meng et al., 2024), these methods can have issues with capturing subtle nuances between different movements. Thus, many researchers still prefer manual scoring for its ability to flexibly judge subject motions for behavior categorization (Hånell and Marklund, 2014).

In this work, we present a novel graphical user interface (GUI) that aims to enhance the rigor and reproducibility of manual scoring of TST without adding appreciable time or work. In brief, the GUI records the mobility status timelines assessed by two independent scorers, identifies periods of disagreement, and enables rescoring of these periods to produce a final, multi-researcher vetted mobility status timeline and associated immobility score. Herein we describe the GUI, and we demonstrate its performance in a cohort of mice subject to closed head injury.

## Materials and Methods

### Animal details

All animal procedures were performed in accordance with the Emory University animal care committee's regulations. Mice were group housed with up to five mice per cage on a 12 h light/dark cycle with lights on from 7 A.M. to 7 P.M. Food and water were given *ad libitum*.

A total of 109 2–5 months old C57BL/6J mice (*n* = 64 male, *n* = 45 female) were used to test the GUI. To demonstrate performance across a wide range of TST times, a subset of 74 mice (34 female, 40 male) were subject to a well-characterized repetitive closed head injury model (Meehan et al., 2012; Buckley et al., 2015; Pybus et al., 2024) consisting of five hits spaced once daily. For the injury model, mice were anesthetized with 3% isoflurane in 100% oxygen for ∼2 min. The anesthetized mouse was positioned beneath a 96 cm vertical guide tube (49035K85, McMaster-Carr) on a Kimwipes task wipe (Kimberly-Clark) and grasped by the base of the tail. Hits were administered by dropping a 54 g bolt down the guide tube such that the bolt impacted the dorsal surface of the head approximately between the coronal and lambdoid sutures. Upon impact, the head broke through the task wipe and experienced a rapid, unrestrained head rotation along the anterior-posterior plane. After injury, mice were closely observed until they regained righting reflex. The remaining 35 mice were subject to sham injury, receiving no head trauma but subject to equivalent anesthesia exposure as the injured animals. TST was conducted 4–17 d after the final closed head injury/sham injury.

### TST protocol

Prior to TST, mice were relocated to a designated testing room to habituate for a minimum of 30 min. Each mouse completed a 6 min TST trial. The TST arena consisted of a metal rod secured to the sides of a 33 cm × 33 cm × 33 cm wooden box. The rod hung parallel to the base, 25 cm from the base of the box. The front and top sides of the box were removed to allow video recording while blocking potential distractions. For the trial, the tail was taped to the metal rod, and a 5 cm long piece of flexible cylindrical plastic tubing (inner diameter = 1 cm) was placed on the mouse's tail to prevent tail climbing attempts.

Videos of TST trials were taken with a Basler acA1300-60gm camera acquired using the EthoVision XT Base Module software (Noldus Information Technology). Each TST trial was independently scored by two different scorers using the GUI described herein. A rescore was conducted on the two independent scores by a final independent scorer using the GUI. A subset of TST trials were also manually scored with the traditional stopwatch method; this scoring was used to assess differences between traditional manual scoring and the GUI. All scorers were instructed that mobility includes tail climbing attempts, running with all four feet, and jolting and twitching movements. Immobility includes hanging without moving, paddling with only two feet, and pendulum swinging from momentum.

### TST score helper GUI overview

**Figure 1.**
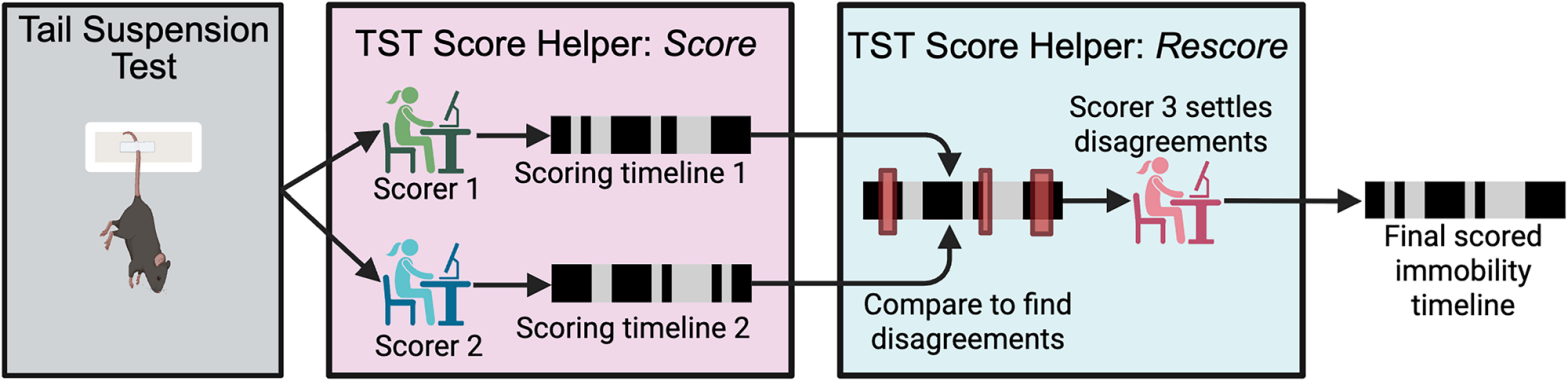
Flow chart depicting the process of using TST Score Helper GUI. After completing the 6 min tail suspension test, a recording of the trial is loaded into the TST Score Helper GUI. First, utilizing the TST Score Helper: Score mode, Scorer 1 and Scorer 2 independently score the trial and generate a scoring timeline of mobility/immobility time segments. Next, utilizing the TST Score Helper: Rescore mode, disagreements between the two scoring timelines are identified (red rectangles) and presented to Scorer 3, who rescores these periods of disagreement. The GUI then produces a final, multi-scorer vetted immobility timeline. Created in BioRender. https://BioRender.com/j39aj6l.

We developed a MATLAB-based GUI to assist with TST scoring, dubbed “TST Score Helper.” The program was written in MATLAB R2024 (MathWorks). It can be run within MATLAB or as a standalone program. An overview of how the TST Score Helper GUI works is shown in Figure 1.

The TST Score Helper GUI has two modes: “score” and “rescore”. Using the score mode, the full video of the TST trial is presented for the scorer to review in real time. The scorer is presented with the video replay and is instructed to toggle a switch that denotes the subject's mobility state. The scoring results are saved as two .csv files. The first file, which we will refer to as the “mobility status table,” records the subject's mobility state over the course of the trial, saved as 5 columns: “Mobility_state”, “Mark_frames”, “Interval_frames”, “Mark_sec”, and “Interval_sec”. Each row of the table represents a continuous segment of the trial with an unchanging mobility state. A new row is created each time the scorer clicks the toggle switch (also referred to as “mark”). The “Mobility_state” column alternates between 0 and 1, denoting immobility and mobility state, respectively. The “Mark_frames” and “Mark_sec” columns denote the frame number and frame time (in seconds), respectively, when the mobility status changed. The “Interval_frames” and “Interval_sec” columns denote the number of frames and the duration (in seconds), respectively, that the subject remained in the given mobility state. The second file consists of the cumulative immobility time, calculated as the sum of the immobility interval durations, the cumulative immobility time after 2 min, and other video specifications including the video file path, video file name, scorer name and date, and path to the score results.

Using the rescore mode, two mobility status tables are loaded and compared for periods of mobility status disagreement. The scorer is presented the periods of disagreement and is instructed to score these sections by choosing a mobility state for the period of disagreement with the toggle switch. The scorer locks in the choice of mobility status for the period of disagreement by selecting next, which also advances the scorer to the next period of disagreement. Upon review of all periods of disagreement, a final mobility status table and a cumulative immobility time are saved to .csv files with the same format as the files described in the previous paragraph; the filename of these files indicates the data was generated with the rescore mode.

### TST score helper GUI: score mode pipeline

1. Upload a video of the TST trial to be scored.
2. Replay the video.
3. Scorer toggles the mobility switch as the subject's mobility status changes.
4. Save the mobility status table and total immobility time .csv files.

### TST score helper GUI: rescore mode pipeline

1. Upload a video of the scored TST trial along with the mobility status tables from 2 independent scorers. Note, tables must be generated via the TST Score Helper program using the score mode. Editing these files manually may result in errors.
2. To account for variations in scorer reaction time between the observation of mobility status change and the time to toggle the button, align the 2 mobility status tables via cross-correlation with the MATLAB function *xcorr*.
3. Generate a mobility timeline vector for each scorer that is the length of the video (in frames). Assign each entry of this vector to either 0 for immobility or 1 for mobility based on the cross-correlated mobility status tables.
4. Identify disagreement clips, defined as times where scorer 1 and scorer 2 mobility timeline vectors have different values. Disagreement clips are determined by adding the two timeseries together and finding all values equal to 1.
5. Present the disagreement clips longer than the selected minimum length to the re-scorer, who watches each clip and then toggles the mobility switch to assign the subject's mobility status for the entire clip.
6. Generate a new mobility timeline vector starting with a copy of the primary scorer's mobility timeline vector. By default, the primary scorer is set to scorer 1, but this selection is changeable on the Settings page. Using the disagreement clips identified in step 4, a rescored mobility status table is produced by using the primary scorer's timeline vector as the basis and replacing any disagreement clips with the newly scored mobility status.
7. Sum the total immobility time of the rescored mobility status table.
8. Save the mobility status table and total immobility time .csv files.

### GUI accessibility

The TST Score Helper GUI can be run as a standalone program or as a MATLAB script. The TST Score Helper MATLAB code (1), installer (2, 3), and template spreadsheets (4, 5) are located at https://github.com/BuckleyLabEmory/TSTScoreHelper. Separate versions of the installer for Windows and Mac are included. If running as a MATLAB script, the Image Processing Toolbox must be installed (MathWorks).

Detailed instructions for software installation and examples are included in 6. Two example videos are included (Movies 1, 2). Each video has two score examples (7–10), which can be used to try the rescore mode.

**Movie 1.** Example video of a TST trial. Example score results for this video are included in 7, 8 to try the rescore mode. [View online]

**Movie 2.** Example video of a TST trial. Example score results for this video are included in 9, 10 to try the rescore mode. [View online]

### TST analysis GUI: how to use

**Figure 2.**
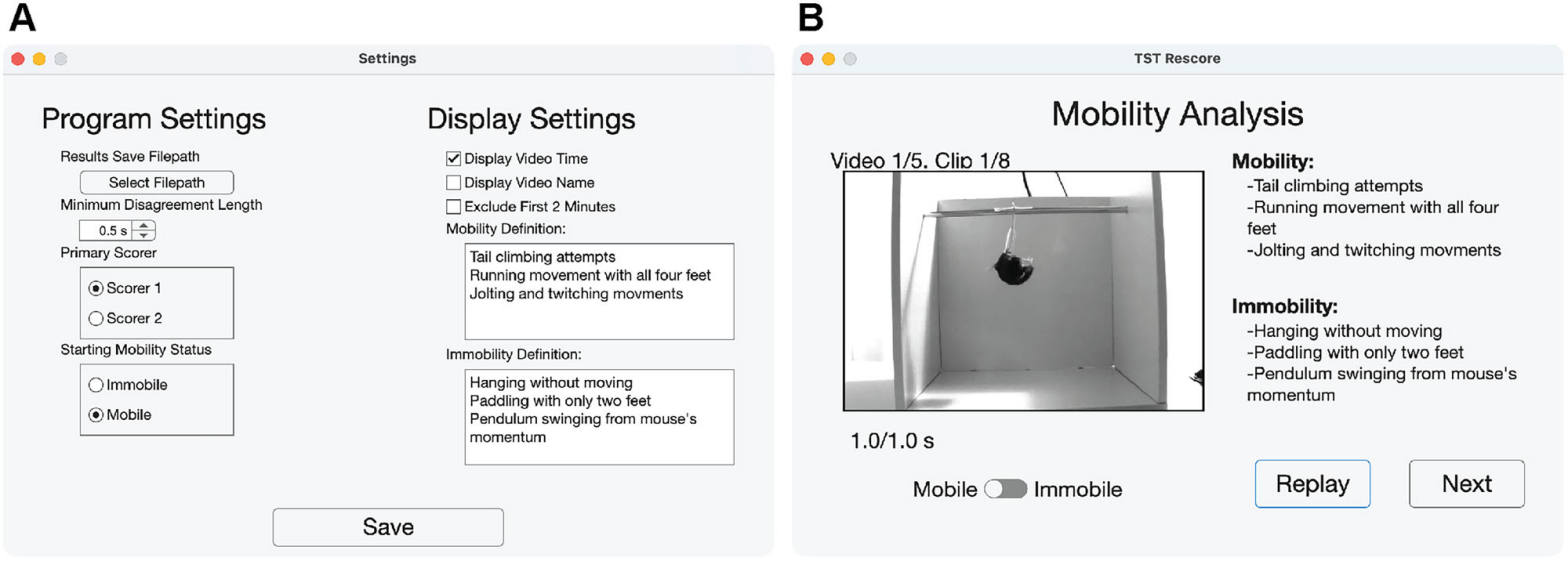
Screenshots of the TST Score Helper GUI's (***A***) settings page and (***B***) rescore page.

The GUI opens on the main menu page. On this page, the user (dubbed the “scorer” herein) selects the scoring type (score entire video or rescore video) and enters their name. The scorer's name is used in the filename for saved results. The main menu page also has a “Settings” button that leads to a settings page (Fig. 2*A*), where the scorer can access program and display settings. Program settings include the file path where results are saved, the minimum disagreement length that triggers rescoring, the primary scorer whose scores are used to fill in nonrescored portions of the trial, and the expected initial mobility status. Display settings include options to display video time and name and to exclude the first 2 min (only applicable to the score mode). Additionally, the definitions for mobility and immobility that are displayed while the trial plays can be edited from the settings page.

On the settings page, the scorer can set the file path to save results and the displayed definitions for mobility and immobility. The scorer can also select whether to make the video time and name hidden (to avoid scoring bias) and whether to include the first 2 min of the trial video. In rescore mode, the minimum disagreement length can be adjusted to change the shortest duration a disagreement clip must be to be rescored.

From the main menu screen, once the scorer selects the “Next” button, the program prompts the user to upload a .csv file containing the names of the TST trial video(s) to be scored. This spreadsheet must follow the format of the provided template spreadsheet for the scoring mode (score or rescore). For score mode, the template spreadsheet is called “example_scoring_filepath_spreadsheet.csv” (4). For rescore mode, the template spreadsheet is called “example_rescoring_filepath_spreadsheet.csv” (5), and it requires the video file paths along with the file paths for the 2 score spreadsheets that have been generated by the GUI in score mode. Once the spreadsheet is uploaded, the scorer can press the “Next” button to score the video. In score mode, the program presents the full video, and the user clicks on the “Mobile/Immobile” switch to change between the two mobility states. The subject's starting mobility state can be changed in the Settings page. Each click on the “Mobile/Immobile” switch records a mark and corresponding video frame number and frame time. In rescore mode, the program presents clips from the video that the 2 scorers disagreed on. For each disagreement clip, the user reviews the clip and makes a final decision on the subject's mobility state by toggling the “Mobile/Immobile” switch (Fig. 2*B*).

Once the full video (score mode) or all disagreement clips (rescore mode) are reviewed, the mobility status table is saved as a .csv file in a folder at the same file path as the video file. The program creates a folder with the same name as the video in the same folder as the video file and saves the spreadsheet in this folder. If this folder already exists, the program does not create a new folder.

### Statistical analysis

In the subset of TST trials for which traditional stopwatch scoring was available, we compared the stopwatch scores to the TST Score Helper GUI scores using Pearson's correlation coefficient, *R*, and associated *p* value along with Bland–Altman analysis. To investigate the reliability of manual scoring, Pearson's correlation coefficient, *R*, between scorer 1's and scorer 2's immobility scores generated from TST Score Helper GUI Score mode was calculated. A Bland–Altman plot was used to visualize the difference between scorer 1's and scorer 2's immobility scores, and the mean bias and limits of agreement were quantified. All statistical analysis was performed in MATLAB 2025a (MathWorks).

## Results

**Figure 3.**
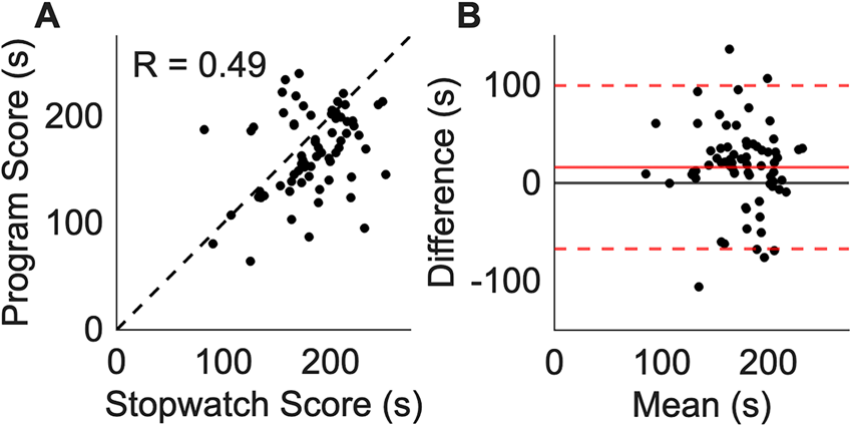
Comparison of scoring using the traditional manual stopwatch method versus the TST Score Helper GUI program in score mode. ***A***, Relationship between immobility times (seconds) assessed by the two different methods on *n* = 71 TST trials. The dashed line denotes the line of unity. ***B***, Bland–Altman plot of the scoring method agreement. The horizontal axis denotes the mean of the two immobility times, and the *y*-axis denotes the difference. The solid black horizontal line denotes *y* = 0. The solid red horizontal line represents the mean difference between the scorers, and the dotted red horizontal lines represent the limits of agreement, which are calculated as 1.96 × standard deviation of the mean difference between scores.

To demonstrate the challenges with traditional stopwatch-based scoring, we compared this approach to the results obtained from the TST Score Helper GUI in score mode. Data was generated by the same scorer (S.E.T.) on *n* = 71 trials. Immobility times generated by the two methods were weakly correlated (*R* = 0.49; Fig. 3*A*) with a mean bias of 16.1 s and limits of agreement from −67.1 to 99.2 s (Fig. 3*B*).

**Figure 4.**
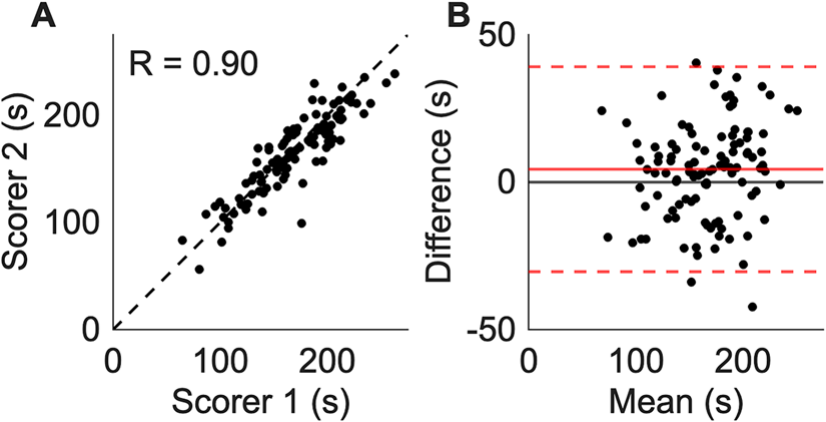
Interscorer reliability of tail suspension test (TST) immobility timing. ***A***, Relationship between the immobility times (seconds) assessed by two independent scorers on *n* = 109 TST trials. The dashed line denotes the line of unity. ***B***, Bland–Altman plot of the interscorer agreement. The horizontal axis denotes the mean between the two scorers’ immobility times and the *y*-axis denotes the difference. The solid black horizontal line denotes *y* = 0. The solid red horizontal line represents the mean difference between the scorers, *y* = 4.4. Dotted red horizontal lines represent the limits of agreement, *y* = −30.4 and *y* = 39.2, which are calculated as 1.96 × standard deviation of the mean difference between scorers.

To further demonstrate the challenges with manual scoring of TST, the TST Score Helper GUI was used in score mode on 109 TST trials scored by two scorers (S.E.T. and P.S.). Scorers were blinded to the other's results. Median (interquartile) immobility times assessed by scorer 1 and scorer 2 were 168.6 (138.7, 198.6) and 169.6 (144.1, 195.1)s, respectively. Upon rescoring, the median (interquartile) immobility time across the cohort was 168.8 s (138.5, 199.2). Total immobility time was strongly correlated between scorers (*R*^2^ = 0.80; Fig. 4*A*) with a mean bias of 4.4 s (Fig. 4*B*). The limits of agreement of the bias extended from −30.4 to 39.2 s (Fig. 4*B*), demonstrating appreciable variability between scorers. Mean immobility time was not correlated with the variability across scorers (calculated as the standard deviation/mean, *p* = 0.84).

**Figure 5.**
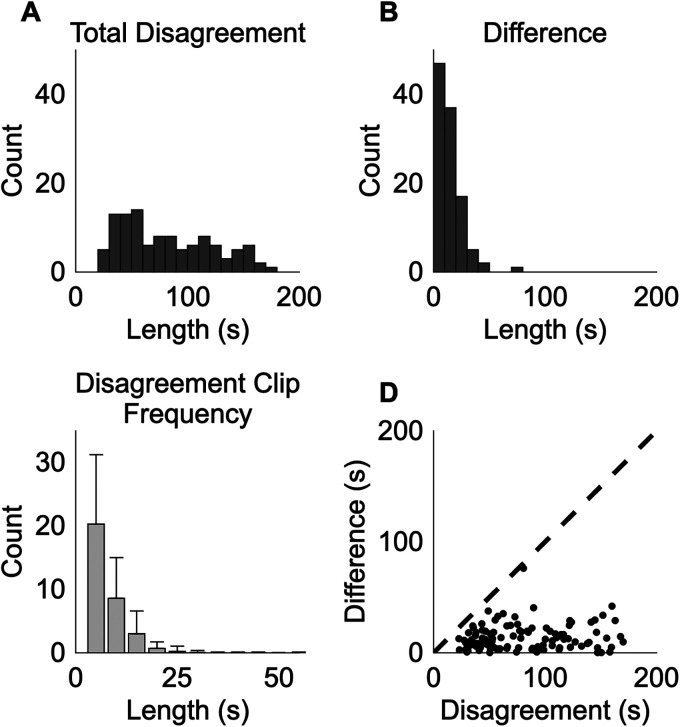
Disagreement between scorers on tail suspension test (TST). For all plots, data from two scorers on 109 TST trials evaluated with the TST Score Helper GUI in score mode were used. ***A***, Histogram of disagreements, i.e., the total length of segments within a TST trial (in seconds) that one scorer assessed as immobile and the other scorer assessed as mobile. ***B***, Histogram of the difference in total immobility time between two scorers. ***C***, Histogram of the number of disagreement clips of duration indicated on the *x*-axis across trials. For each 5 s bin, the bar represents the mean number of disagreement clips in that length range across all trials, and the whiskers represent one standard deviation above the mean. ***D***, Scatterplot of disagreements versus the difference in total immobility time between two scorers. The dashed line denotes the line of unity.

Figure 5 demonstrates that the difference in total immobility time between scorers plotted in Figure 4*B* does not fully reflect the disagreements in the scoring analysis. Figure 5*A* shows a histogram of the total disagreement within each TST video, defined as the total length (in seconds) of segments within a trial that one scorer assessed as immobile and the other scorer assessed as mobile. The total disagreement duration ranges in length between 22.5 and 170.0 s with a mean value of 82.0 s (Fig. 5*B*), which is substantially larger than the mean bias in total immobility time of 4.4 s from Figure 4*B*. Figure 5*C* shows the distribution of disagreement clip lengths for each of the 109 trials. This distribution shows that most disagreements are relatively short in duration, typically <10 s; however, they can be appreciable, with some disagreement clips as longer than 40 s. As shown in Figure 5*D*, no correlation was observed between each trial's total disagreement duration and the difference in total immobility time between scorers.

**Figure 6.**
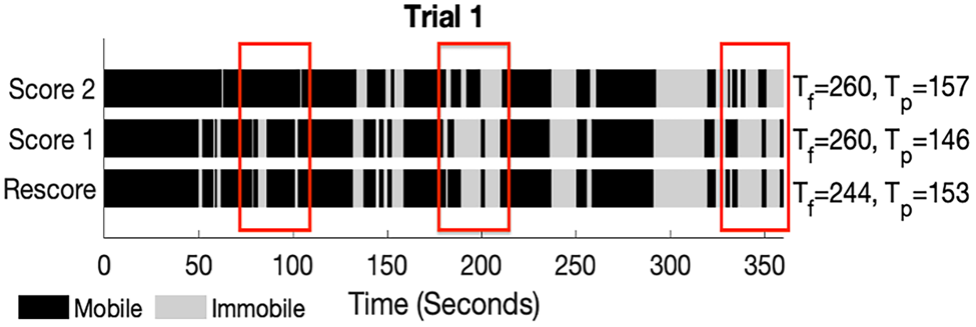
Example timeline produced with the TST Score Helper GUI rescore mode. $T_{f}$ is the full immobility time and $T_{p}$ is the partial immobility time, which only includes immobility time after the first 2 min. The red rectangles surround areas of scorer disagreement.

Figure 6 shows a representative example of the timeline figure generated after rescoring with the TST Score Helper GUI. The GUI plots the mobility status table as a timeline along with the full immobility time, *T_f_*, and the partial (excluding the first 2 min) immobility time, *T_p_*, for each scorer. As is evident from the figure, there were numerous periods of disagreement between the scorers that were resolved upon rescoring. In this example, scorer 1 and scorer 2 both arrived at a total immobility time of 260 s; however, the areas they disagreed on contained more mobile time such that after rescoring, the total immobility time was 244 s.

## Discussion

In this work we demonstrate issues with manual scoring of the tail suspension test that hamper rigor and reproducibility. We develop a MATLAB-based graphical user interface to reduce sources of manual scoring error, thereby improving the fidelity of the results. This program is free, easy to use, and open source.

The benefit of the TST Score Helper GUI is twofold: first, the opportunity for errors is reduced through digitization of the scoring process, and second, the rescoring functionality enables users to identify and correct scoring disagreements. With regard to the first benefit, we note that TST scoring is traditionally performed by a scorer with a stopwatch who manually times mobility while viewing the video of the TST trial. Our GUI links the trial video time to the scoring timer, thereby removing difficulties with managing the video player and timer concurrently. While human reaction time still contributes to analysis error, the GUI significantly minimizes opportunities for human error. The GUI also allows for pausing the trial video and timer simultaneously to eliminate errors caused by uncoupling of video and timer times. With regard to the second benefit, it is clear from our results (Figs. 5, 6) that even when the scorers’ total immobility times are similar, the total disagreement duration may be appreciable (Figs. 4, 5). Periods of differing mobility score provide an easy target for error minimization. The GUI allows the user to identify these differing periods and rescore them for improved fidelity of the results.

This approach is not without limitations. First, the approach requires considerable user time; the GUI requires two independent scorers to complete the initial scoring, and then a third scorer (ideally not one of the original scorers) must review periods of disagreement to complete the rescore. Second, the rescore functionality only catches periods of disagreement, and it cannot directly detect errors in classification of mobility status. If both scorers erroneously categorized the mouse as mobile when it was immobile, the GUI would not identify this error. Finally, periods of disagreement shorter than the user-defined minimum disagreement length default to the primary scorer, which can introduce an opportunity for bias. The minimum disagreement length should be chosen to minimize this bias while excluding periods of disagreement that are too short to reliably determine mobility status.

### Conclusion

Herein we show challenges with manual scoring of the tail suspension test that hamper rigor and reproducibility. Nuanced behavioral categorization leading to complex scoring criteria and lengthy trial time requiring extended focus, both increase opportunity for errors during manual scoring. We present a novel graphical user interface to reduce sources of manual scoring error and improve the fidelity of results. Future work should explore the combination of this software with artificial intelligence and/or machine learning algorithms trained on large TST datasets to implement an entirely automated scoring method that further minimizes scorer error.

10.1523/ENEURO.0318-25.2026.d1 Data 1 TSTScoreHelper MATLAB script. Download Data 1, ZIP file.

10.1523/ENEURO.0318-25.2026.d2 Data 2 Standalone program installer for Windows. Download Data 2, ZIP file.

10.1523/ENEURO.0318-25.2026.d3 Data 3 Standalone program installer for Mac. Download Data 3, ZIP file.

10.1523/ENEURO.0318-25.2026.d4 Data 4 Template spreadsheet for score mode. Download Data 4, CSV file.

10.1523/ENEURO.0318-25.2026.d5 Data 5 Template spreadsheet for rescore mode. Download Data 5, CSV file.

10.1523/ENEURO.0318-25.2026.d6 Data 6 Detailed instructions for installation and use with examples. Download Data 6, DOCX file.

10.1523/ENEURO.0318-25.2026.d7 Data 7 Example score result 1 for example video 1 (Movie 1). Download Data 7, CSV file.

10.1523/ENEURO.0318-25.2026.d8 Data 8 Example score result 2 for example video 1 (Movie 1). Download Data 8, CSV file.

10.1523/ENEURO.0318-25.2026.d9 Data 9 Example score result 1 for example video 2 (Movie 2). Download Data 9, CSV file.

10.1523/ENEURO.0318-25.2026.d10 Data 10 Example score result 2 for example video 2 (Movie 2). Download Data 10, CSV file.

## Synthesis

Reviewing Editor: Adrien Peyrache, McGill University Decisions are customarily a result of the Reviewing Editor and the peer reviewers coming together and discussing their recommendations until a consensus is reached. When revisions are invited, a fact-based synthesis statement explaining their decision and outlining what is needed to prepare a revision will be listed below. The following reviewer(s) agreed to reveal their identity: Chandrashekhar Borkar, Sofia Skromne Carrasco.

The present study introduces TST Score Helper, a MATLAB-based GUI designed to enhance rigor and reproducibility in scoring the tail suspension test (TST) in rodents. The tool addresses key limitations of manual stopwatch-based scoring and of automated systems that often fail to capture nuanced behaviors such as swinging or partial limb paddling. A dedicated "rescore" function compares results from two independent scorers, flags discrepancies, and enables a third reviewer to resolve disagreements. This open-source approach promotes transparency, reduces timing errors, and increases the reliability of manual scoring.

While the contribution is practical and timely, several issues should be addressed to make the study publication-ready. The tool's design remains labor-intensive, requiring three human scorers, and scalability is limited compared to fully automated pipelines. Moreover, the claim that automated methods cannot distinguish complex movement patterns such as body versus limb motion is not fully demonstrated; tools such as DeepLabCut have achieved this. The present work should therefore be framed as a transitional step toward future integration of AI or computer-vision-based scoring.

Major Comments

1) The source code should be deposited on a public repository (e.g., GitHub) with example datasets, sample videos, and scoring templates provided as to facilitate reproducibility.

2) The tool could not successfully load .mp4 videos in either the Windows version or the MATLAB GUI. The reported error was "Unrecognized function or variable 'video_panel_height'", even after installation of the Image Processing Toolbox. An error sound was triggered without a displayed message, making troubleshooting difficult. These issues should be debugged, and clearer instructions provided for proper file loading.

3) Disagreement clips shorter than the minimum clip length are automatically resolved using Scorer 1's results. This behavior may introduce bias, especially for high minimum-length thresholds. A user option to select which scorer's data are used, or a clear on-screen indication of this default, would improve transparency.

4) A direct comparison of immobility scoring obtained manually versus with the help of TST Score Helper would demonstrate how the tool enhances reproducibility.

5) Although scoring variability was intentionally introduced to capture a broad range of immobility levels, no quantitative results (e.g., trauma vs. sham) are provided. Assessing whether scoring variability correlates with immobility percentage would strengthen the study.

6) Scoring always begins with a mobile interval, which may not apply to highly immobile or sedated animals. Implementing an initial-state selector or a short calibration segment would increase flexibility.

7) Timelines are stored at millisecond resolution, which exceeds video frame-rate precision. Frame-based indexing would be more appropriate and less misleading.

8) Current exports are limited to Excel. Providing standardized output formats such as CSV or JSON with metadata would improve interoperability with downstream analysis pipelines and enhance the open-source value of the tool.

9) Including a simple flowchart illustrating the scoring → comparison → rescoring pipeline would clarify the tool's logic and usability.

Minor Comments

1) Only one paper (Nandi, 2021) is cited when discussing limitations of automated scoring. Additional relevant work such as Juszczak et al. (2006) and Meng et al. (2024) should be included.

2) Line 48: "nuisances" should read "nuances."

3) Figure 3C: Replace "Frequency" with "Disagreement Clip Duration" for accuracy. Means and error bars should be made more visually distinct, for example by using circles for means.

5) Lines 123 and 130: "TST Score Helper GUI: Score" and "TST Score Helper Software: Rescore" should be harmonized if the functionality is equivalent.

6) Typo correction: "speadsheet" → "spreadsheet."

7) In the Settings menu, "Minimum Clip Length" could be renamed "Minimum Disagreement Length" for clarity.

References:

Juszczak, G. R., Sliwa, A. T., Wolak, P., Tymosiak-Zielinska, A., Lisowski, P., &Swiergiel, A. H. (2006). The usage of video analysis system for detection of immobility in the tail suspension test in mice. Pharmacology, biochemistry, and behavior, 85(2), 332-338. https://doi.org/10.1016/j.pbb.2006.08.016

Meng, X., Xia, Y., Liu, M., Ning, Y., Li, H., Liu, L., &Liu, J. (2024). A deep-learning-based threshold-free method for automated analysis of rodent behavior in the forced swim test and tail suspension test. Journal of neuroscience methods, 409, 110212. https://doi.org/10.1016/j.jneumeth.2024.110212

## Author Response

We would like to thank our reviewers for their constructive feedback. We are grateful for their thorough review of our work, and we believe addressing these comments has considerably strengthened the manuscript. Below we restate each reviewer comment and provide a detailed response.

1) The source code should be deposited on a public repository (e.g., GitHub) with example datasets, sample videos, and scoring templates provided as to facilitate reproducibility.

Response: We will store the source code, example datasets, sample videos, and scoring templates on GitHub once the manuscript is published.

2) The tool could not successfully load .mp4 videos in either the Windows version or the MATLAB GUI. The reported error was "Unrecognized function or variable 'video_panel_height'", even after installation of the Image Processing Toolbox. An error sound was triggered without a displayed message, making troubleshooting difficult. These issues should be debugged, and clearer instructions provided for proper file loading.

Response: Thank you so much for catching this bug. We have fixed this issue, and we have revised the code throughout to display error messages. We also have added detailed instructions, Instructions_and_Examples.docx, for proper file loading in 3.

3) Disagreement clips shorter than the minimum clip length are automatically resolved using Scorer 1's results. This behavior may introduce bias, especially for high minimum-length thresholds. A user option to select which scorer's data are used, or a clear on-screen indication of this default, would improve transparency.

Response: We think this is a great suggestion, and we now implement this functionality. We also comment in the manuscript Discussion about this potential source of bias.

Text change: Lines 279-283 "...periods of disagreement shorter than the user defined minimum disagreement length default to the primary scorer, which can introduce an opportunity for bias. The minimum disagreement length should be chosen to minimize this bias while excluding periods of disagreement that are too short to determine mobility status." 4) A direct comparison of immobility scoring obtained manually versus with the help of TST Score Helper would demonstrate how the tool enhances reproducibility.

Response: We have now added a comparison of manual immobility scoring with a stopwatch to that obtained from the TST Score Helper GUI in score mode(Figure 3). This figure highlights the poor agreement between approaches.

5) Although scoring variability was intentionally introduced to capture a broad range of immobility levels, no quantitative results (e.g., trauma vs. sham) are provided. Assessing whether scoring variability correlates with immobility percentage would strengthen the study.

Response: We investigated the correlation between scoring variability and immobility from the data presented in Figure 4. No correlation was found (p = 0.84). We now include this result in the manuscript.

Text change: Lines 230-231 "Mean immobility time was not correlated with the variability across scorers (calculated as the standard deviation/mean, p = 0.84)." 6) Scoring always begins with a mobile interval, which may not apply to highly immobile or sedated animals. Implementing an initial-state selector or a short calibration segment would increase flexibility.

Response: This is a good suggestion, and we now have added in the functionality for the user to select the initial state.

7) Timelines are stored at millisecond resolution, which exceeds video frame-rate precision. Frame-based indexing would be more appropriate and less misleading.

Response: We have now fixed this timing resolution in our analysis. Timelines are now stored with frame rate precision and use frame based indexing.

8) Current exports are limited to Excel. Providing standardized output formats such as CSV or JSON with metadata would improve interoperability with downstream analysis pipelines and enhance the open-source value of the tool.

Response: We agree and now changed the default file format to csv.

9) Including a simple flowchart illustrating the scoring → comparison → rescoring pipeline would clarify the tool's logic and usability.

Response: We agree with the reviewer's suggestion, and we now include a flowchart as Figure 1.

1) Only one paper (Nandi, 2021) is cited when discussing limitations of automated scoring. Additional relevant work such as Juszczak et al. (2006) and Meng et al. (2024) should be included.

Response: Thank you for pointing us to these references, we now include these and others.

Text change: Lines 45-48 "While promising automated scoring methods combatting these issues have emerged (Juszczak et al., 2006; Bohnslav. 2021; Nandi, 2021; Meng et al., 2024; Isik &Unal, 2023), these methods can have issues with capturing subtle nuances between different movements." 2) Line 48: "nuisances" should read "nuances."

Response: Thank you for catching this typo, we have corrected this error in the revised text.

3) Figure 3C: Replace "Frequency" with "Disagreement Clip Duration" for accuracy. Means and error bars should be made more visually distinct, for example by using circles for means.

Response: The title of Figure 3C is "Disagreement Clip Frequency" and we have modified the caption text for clarity. Additionally, we have modified figure 3C to make the data more visually distinct.

Text change: Figure 5 caption "Histogram of the mean number of disagreement clips within a given trial of a given length." 5) Lines 123 and 130: "TST Score Helper GUI: Score" and "TST Score Helper Software: Rescore" should be harmonized if the functionality is equivalent.

Response: We have corrected this nomenclature throughout the manuscript. The text now reads "TST Score Helper GUI in score mode" and " TST Score Helper GUI in rescore mode".

6) Typo correction: "speadsheet" → "spreadsheet."

Response: Thank you for catching this typo, we have corrected this error in the revised code.

7) In the Settings menu, "Minimum Clip Length" could be renamed "Minimum Disagreement Length" for clarity.

Response: We agree with the reviewer's suggestion and have made this change.

References:

Juszczak, G. R., Sliwa, A. T., Wolak, P., Tymosiak-Zielinska, A., Lisowski, P., &Swiergiel, A. H. (2006). The usage of video analysis system for detection of immobility in the tail suspension test in mice. Pharmacology, biochemistry, and behavior, 85(2), 332-338. https://nam11.safelinks.protection.outlook.com/?url=https%3A%2F%2Fdoi.org%2F10.1016%2Fj.pbb.2006.08.016%26data=05%7C02%7Cerin.buckley%40emory.edu%7Cd6c1a8112b774d68dba008de067668d6%7Ce004fb9cb0a4424fbcd0322606d5df38%7C0%7C0%7C638955302800591684%7CUnknown%7CTWFpbGZsb3d8eyJFbXB0eU1hcGkiOnRydWUsIlYiOiIwLjAuMDAwMCIsIlAiOiJXaW4zMiIsIkFOIjoiTWFpbCIsIldUIjoyfQ%3D%3D%7C0%7C%7C%7C%26sdata=hnR5lzlt1l9MKX70sljRjFBdDKeAf3081IdldRY9FIc%3D%26reserved=0 Meng, X., Xia, Y., Liu, M., Ning, Y., Li, H., Liu, L., &Liu, J. (2024). A deep-learning-based threshold-free method for automated analysis of rodent behavior in the forced swim test and tail suspension test. Journal of neuroscience methods, 409, 110212. https://nam11.safelinks.protection.outlook.com/?url=https%3A%2F%2Fdoi.org%2F10.1016%2Fj.jneumeth.2024.110212%26data=05%7C02%7Cerin.buckley%40emory.edu%7Cd6c1a8112b774d68dba008de067668d6%7Ce004fb9cb0a4424fbcd0322606d5df38%7C0%7C0%7C638955302800609024%7CUnknown%7CTWFpbGZsb3d8eyJFbXB0eU1hcGkiOnRydWUsIlYiOiIwLjAuMDAwMCIsIlAiOiJXaW4zMiIsIkFOIjoiTWFpbCIsIldUIjoyfQ%3D%3D%7C0%7C%7C%7C%26sdata=cJbTQzVrlVg1TFHCYmhc%2B%2F1w5ilz7bq3VefEvXqtC44%3D%26reserved=0
