# Supplementary figures and images for: TST Score Helper: An Open-Source Graphical User Interface for Assisted Manual Scoring of the Tail Suspension Test
Source: eNeuro. 2026 Apr 8;13(4):ENEURO.0318-25.2026. doi: 10.1523/ENEURO.0318-25.2026 (PMC13080431; doi:10.1523/ENEURO.0318-25.2026)

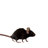

Supplement: Data 3 — Standalone program installer for Mac. Download Data 3, ZIP file. [file eneuro-13-ENEURO.0318-25.2026-s006.zip › TSTScoreHelperInstallerMac.app/Contents/Resources/icon_48.png]

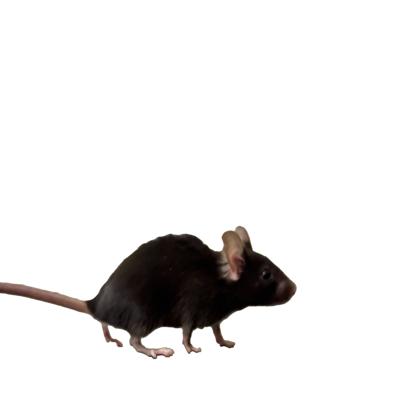

Supplement: Data 3 — Standalone program installer for Mac. Download Data 3, ZIP file. [file eneuro-13-ENEURO.0318-25.2026-s006.zip › TSTScoreHelperInstallerMac.app/Contents/Resources/splash.png]

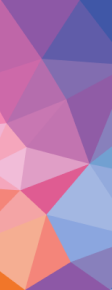

Supplement: Data 3 — Standalone program installer for Mac. Download Data 3, ZIP file. [file eneuro-13-ENEURO.0318-25.2026-s006.zip › TSTScoreHelperInstallerMac.app/Contents/Resources/temp_default_logo.png]
