# Supplementary material for: TST Score Helper: An Open-Source Graphical User Interface for Assisted Manual Scoring of the Tail Suspension Test
Source: eNeuro. 2026 Apr 8;13(4):ENEURO.0318-25.2026. doi: 10.1523/ENEURO.0318-25.2026 (PMC13080431; doi:10.1523/ENEURO.0318-25.2026)
Supplement: Data 6 — Detailed instructions for installation and use with examples. Download Data 6, DOCX file. [file eneuro-13-ENEURO.0318-25.2026-s009.docx]

Table of Contents

[Standalone Software Installation: Windows 2](#_Toc216098358)

[Standalone Software Installation: Mac 6](#_Toc216098359)

[TST Score Helper GUI in score mode: Example 12](#_Toc216098360)

[TST Score Helper GUI in rescore mode: Example 18](#_Toc216098361)

# **Standalone Software Installation: Windows**

1. Double click the file *TSTScoreHelperInstallerWindows* to run. When the Installer window opens, press “Next”.


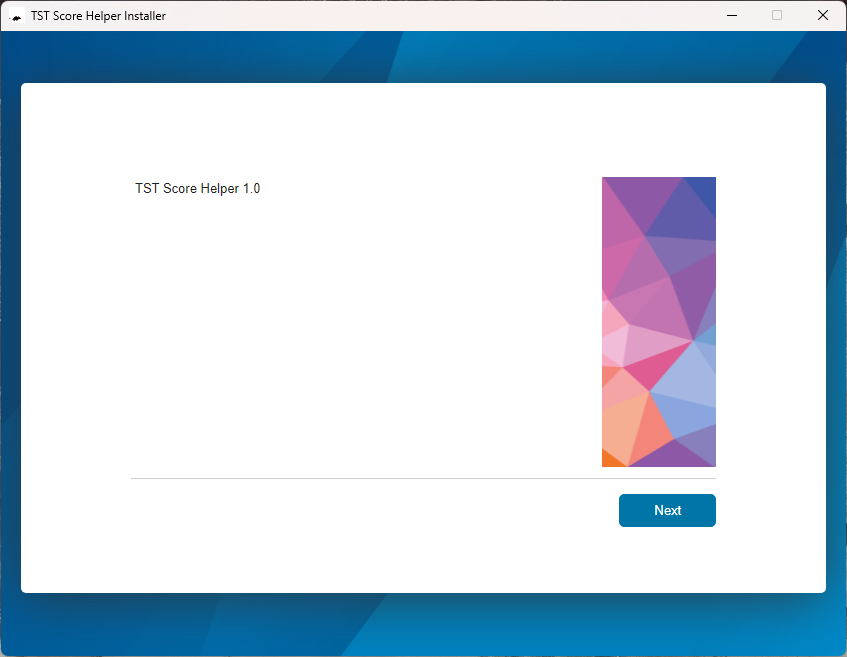


1. Select where the program will be downloaded, and press “Next”.


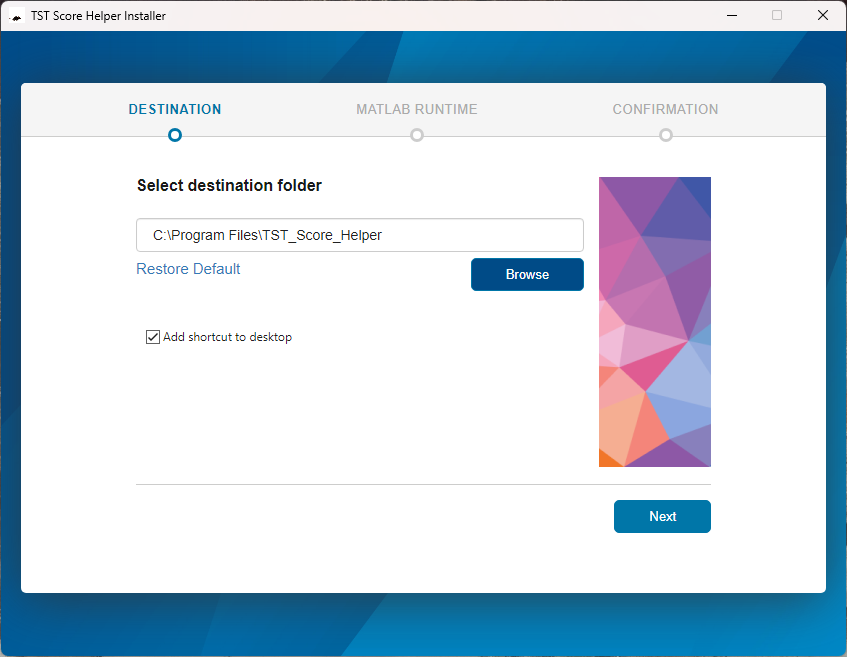


1. If MATLAB Runtime is not installed on your computer, the program will install it. Select where MATLAB Runtime will be downloaded, and press “Next”.


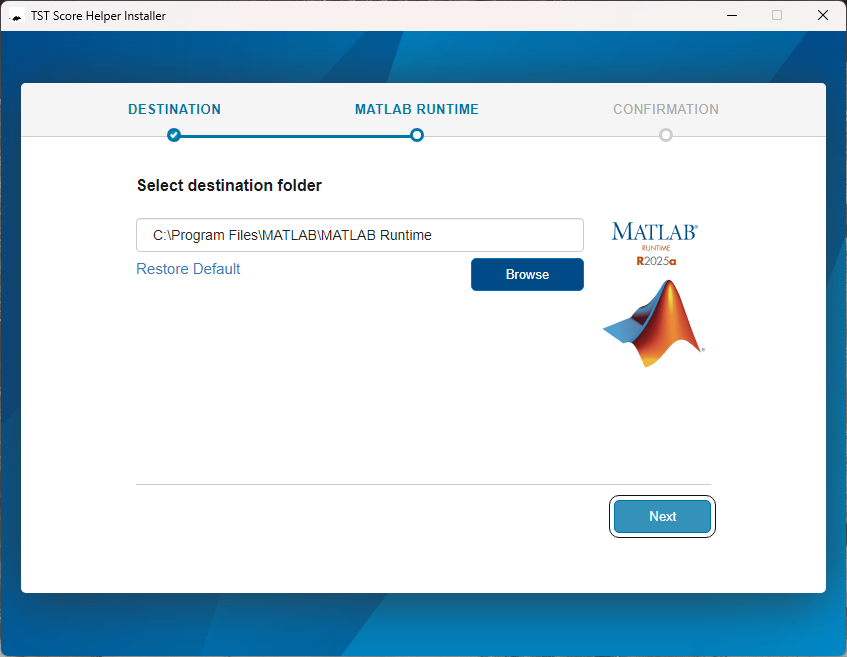


- 1. When prompted about accepting the terms of the license agreement, select “Yes”, and press “Next”.


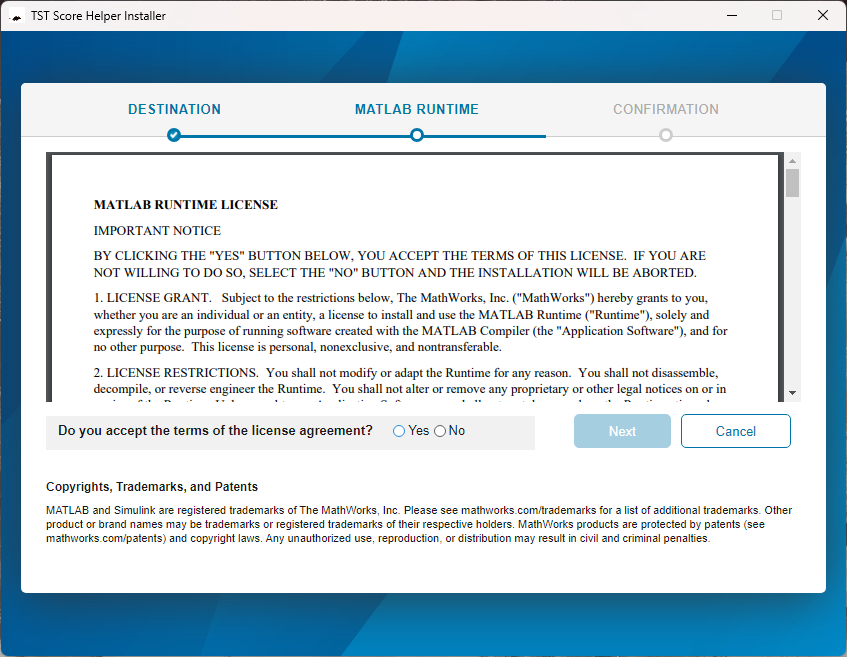


1. Click “Begin installation”.


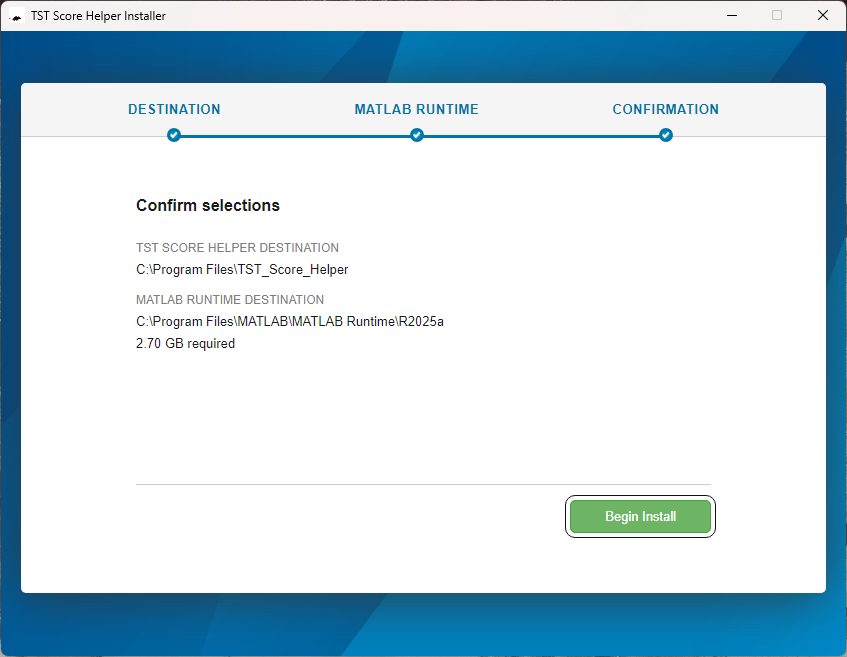


1. Wait for the program to be installed.


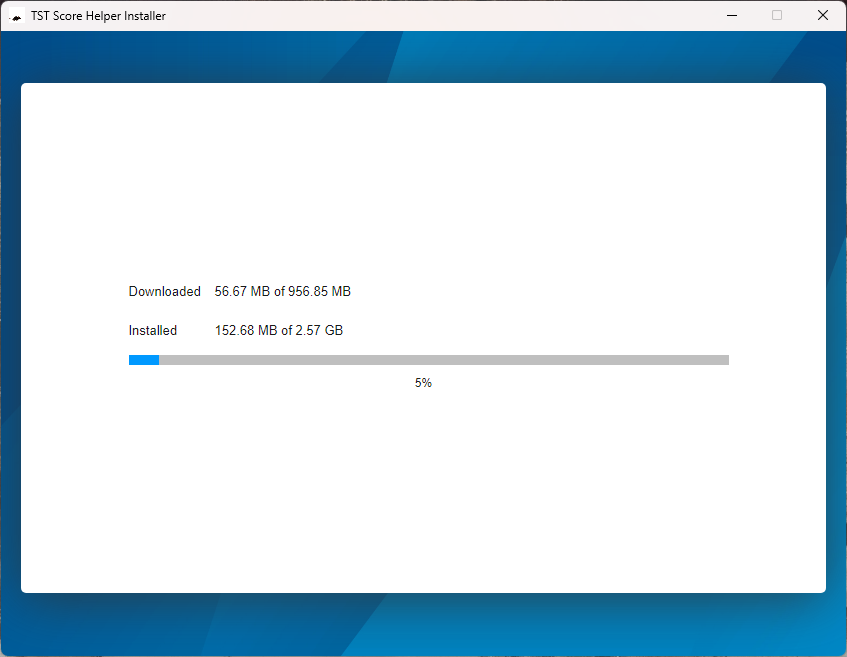


1. Installation is complete. Press “Close” to close the installation window.


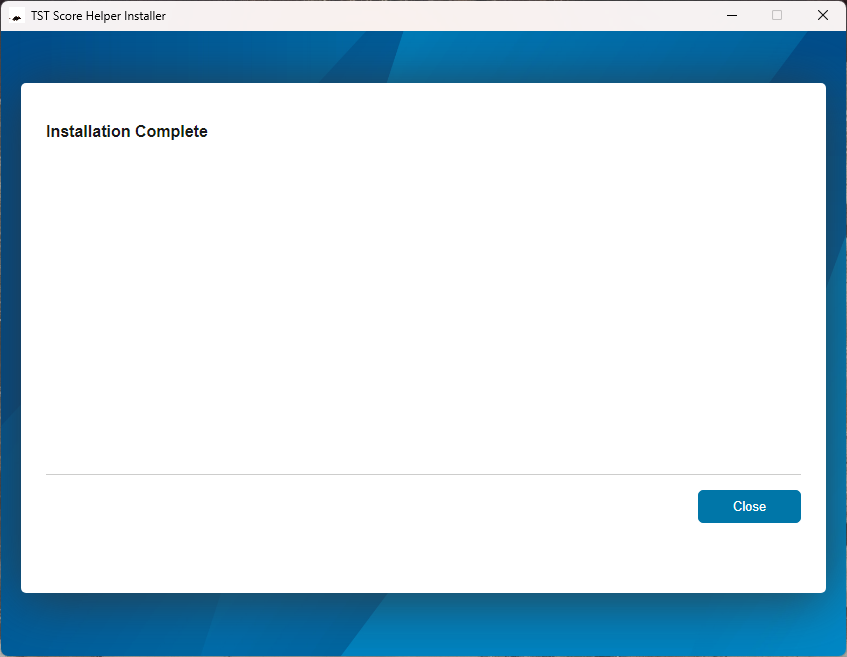


1. Open the TST Score Helper GUI by double clicking on the file *TSTScoreHelper*. The app will take an extra few minutes to open the first time it runs.

# **Standalone Software Installation: Mac**

1. Double click the file *TSTScoreHelperInstallerMac* to run. When the Installer window opens, press “Next”.


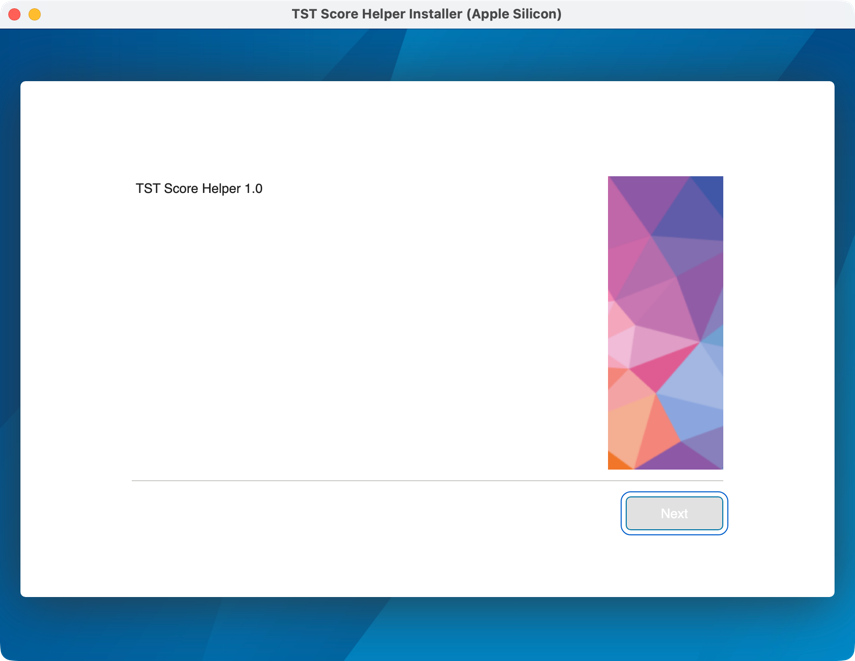


1. Select where the program will be downloaded and press “Next”.


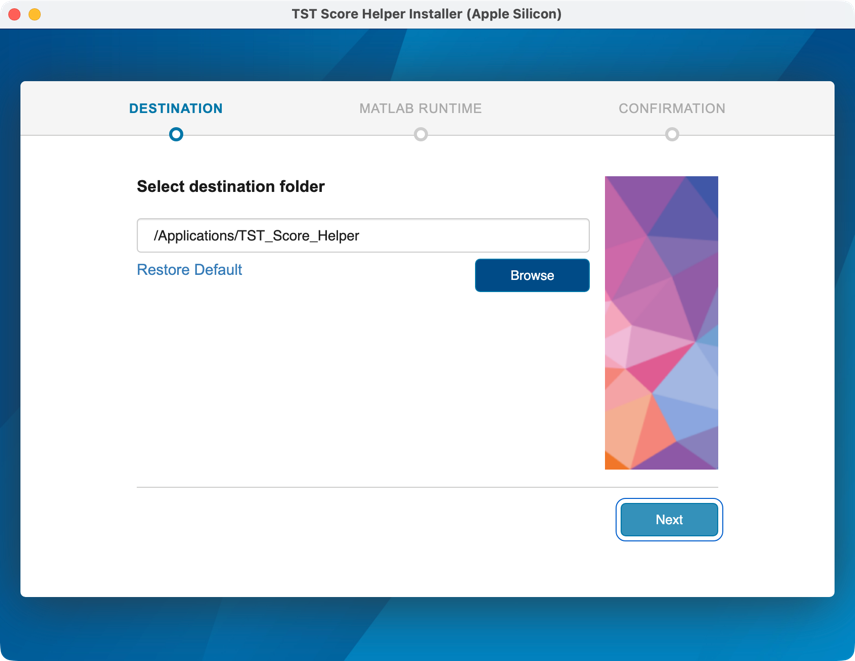


1. If MATLAB Runtime is not installed, the program will install it. When prompted about accepting the terms of the license agreement, select “Yes” and press “Next”.


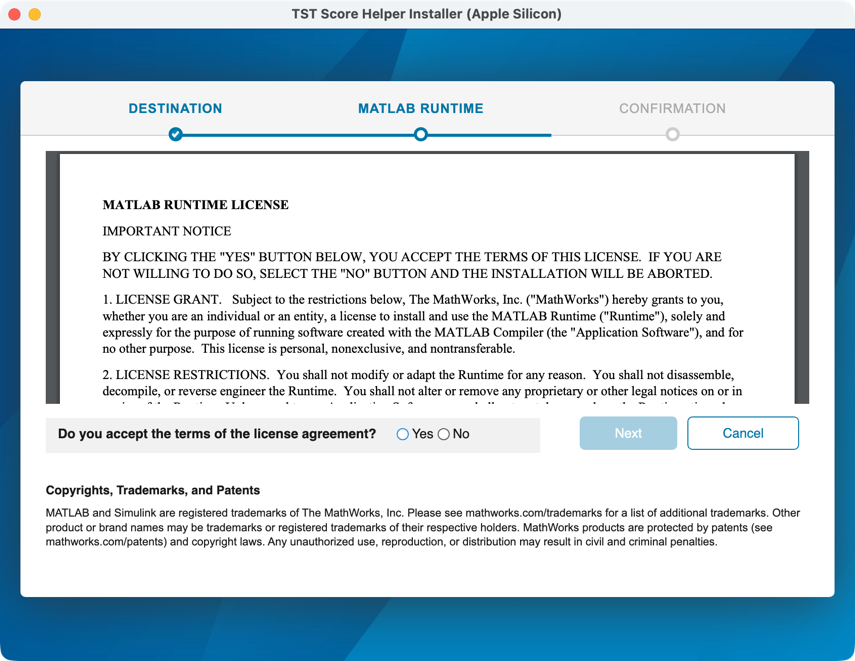


- 1. Select where MATLAB Runtime will be downloaded, and click “Next”.


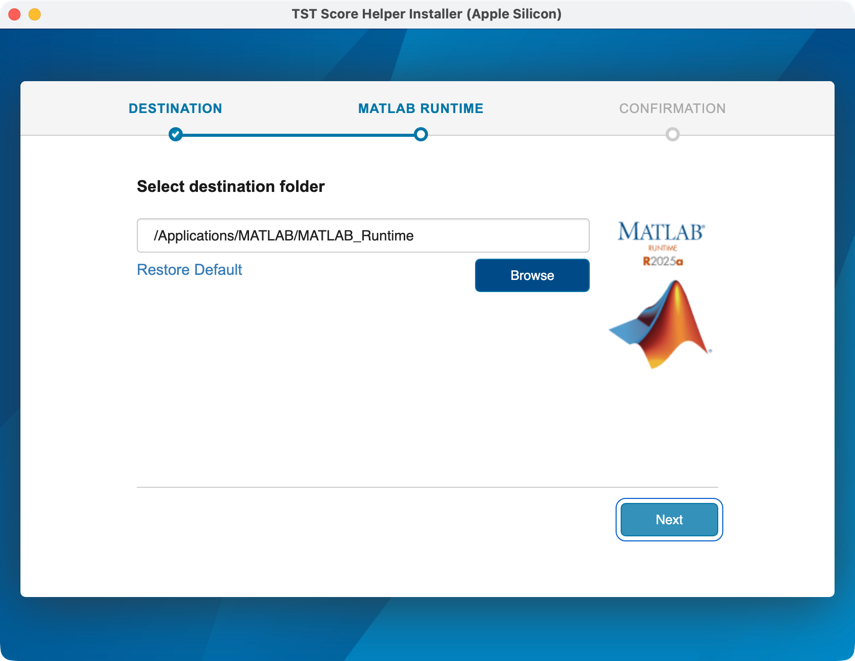


1. Click “Begin installation”.


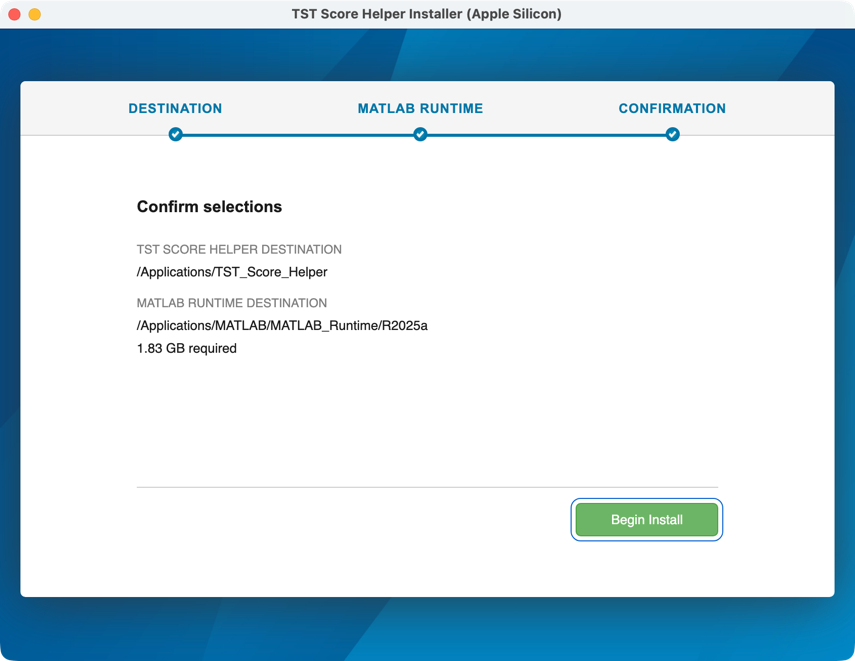


1. Wait for the program to be installed.


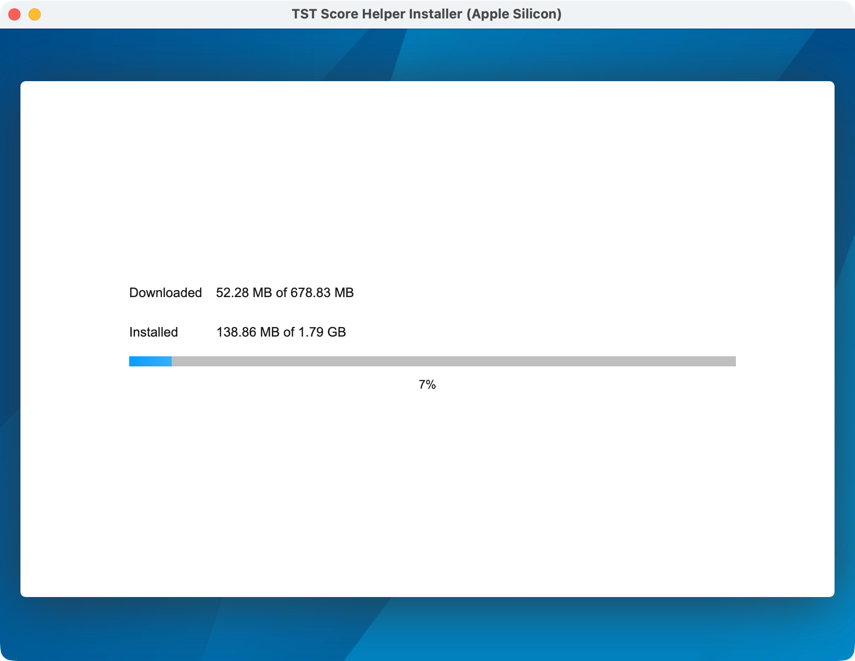


1. Installation is complete. If you are prompted to complete additional configuration steps (shown below), follow steps i-vi. These additional steps occur because of the security feature System Integrity Protection on macOS.


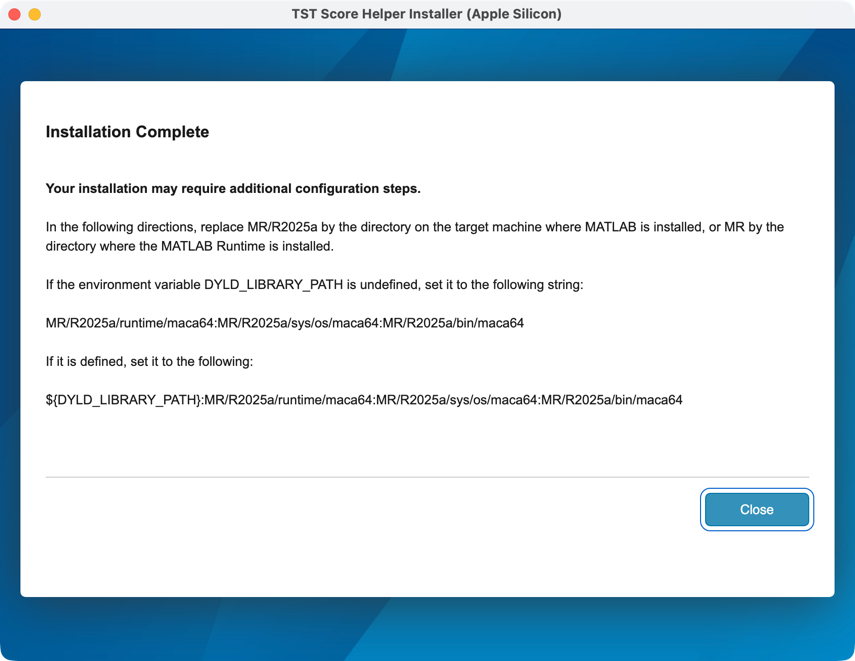


- 1. Open a new Finder window, and navigate to where TST Score Helper is downloaded.


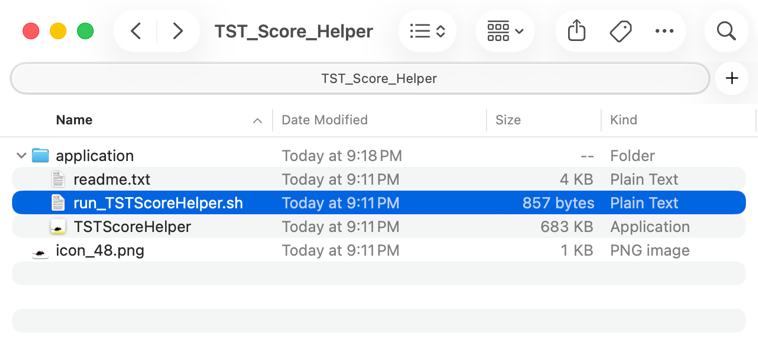


- 1. Double click the file *run_TSTScoreHelper.sh* to open with the TextEdit Application. The file shown below should pop up.


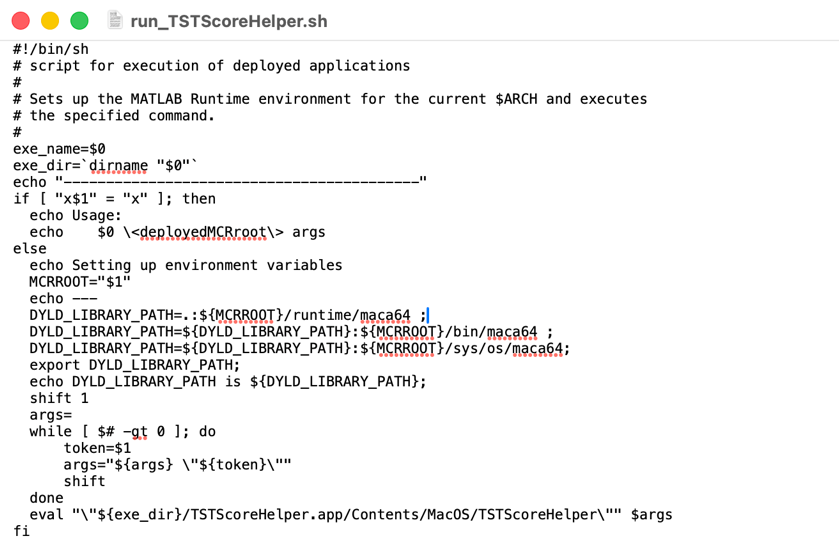


- 1. Locate the 3 lines that start with “DYLD_LIBRARY_PATH=”, marked by the red box in the image below.


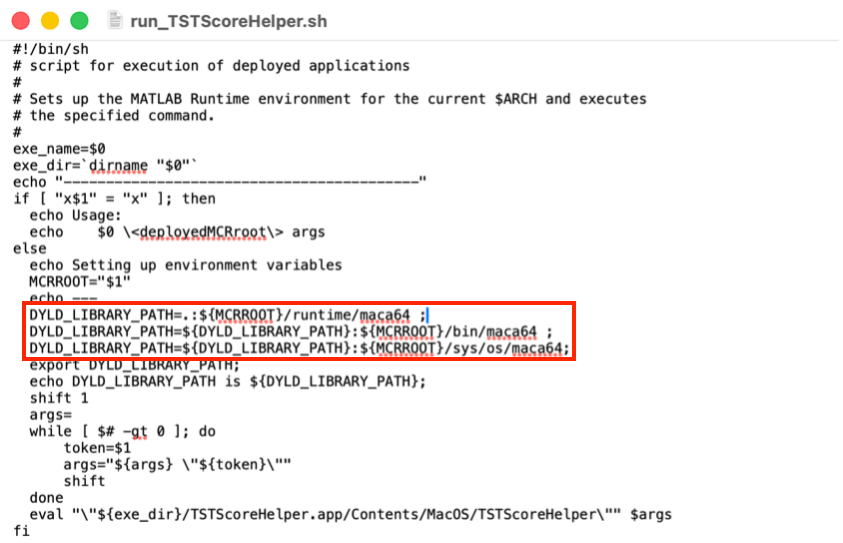


- 1. In the first line, replace the text is between the “.:” and “/runtime…” with the filepath for MATLAB_Runtime, which was set in Steps 3i and 4.


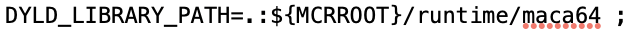


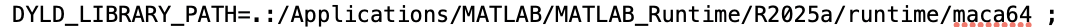


- 1. In the second line, replace the text between the “${DYLD_LIBRARY_PATH}:” and “/bin…” with the filepath for MATLAB_Runtime, which was set in Steps 3i and 4.


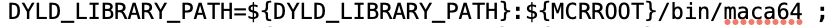


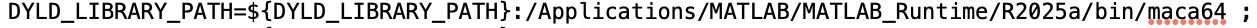


- 1. In the third line, replace the text between the “${DYLD_LIBRARY_PATH}:” and “/sys…” with the filepath for MATLAB_Runtime, which was set in Steps 3i and 4.


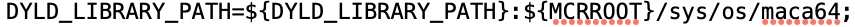


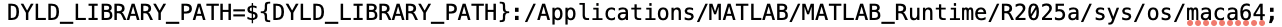


1. Save the file and close.
2. Open the TST Score Helper GUI by double clicking on the file *TSTScoreHelper*. The app will take an extra few minutes to open the first time it runs.

# **TST Score Helper GUI in score mode: Example**

1. Open the software by double clicking on the file *TSTScoreHelper* (if running stand-alone software) or by running the TSTScoreHelper_FullCode.m script in the MATLAB command window (if running on MATLAB). The home page shown below should pop up.


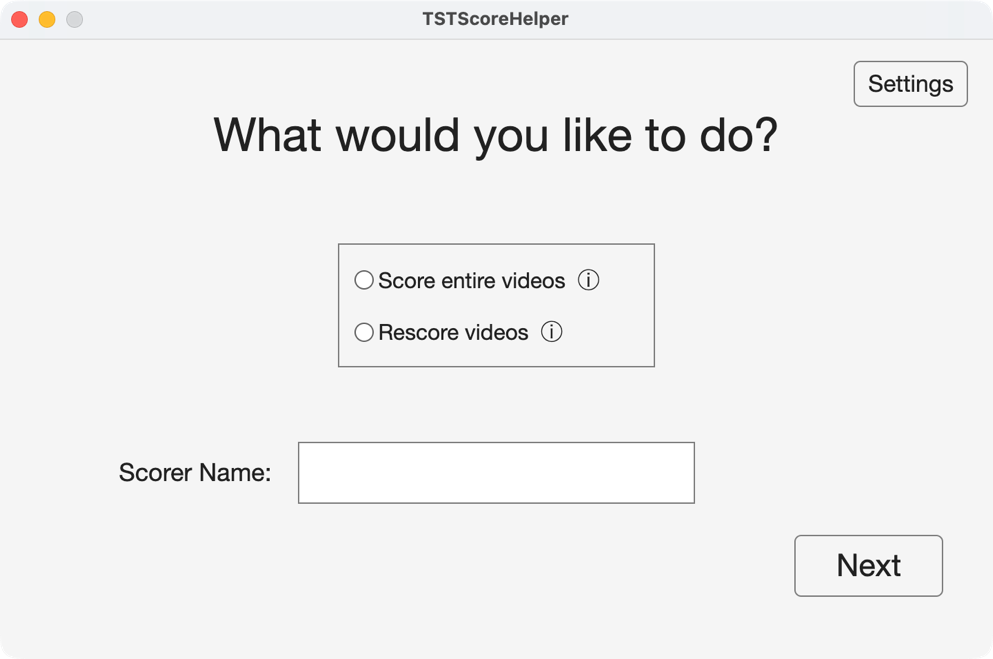


1. Press “Settings”. Settings should be adjusted every time the program is opened. The settings page with the default settings is shown below.


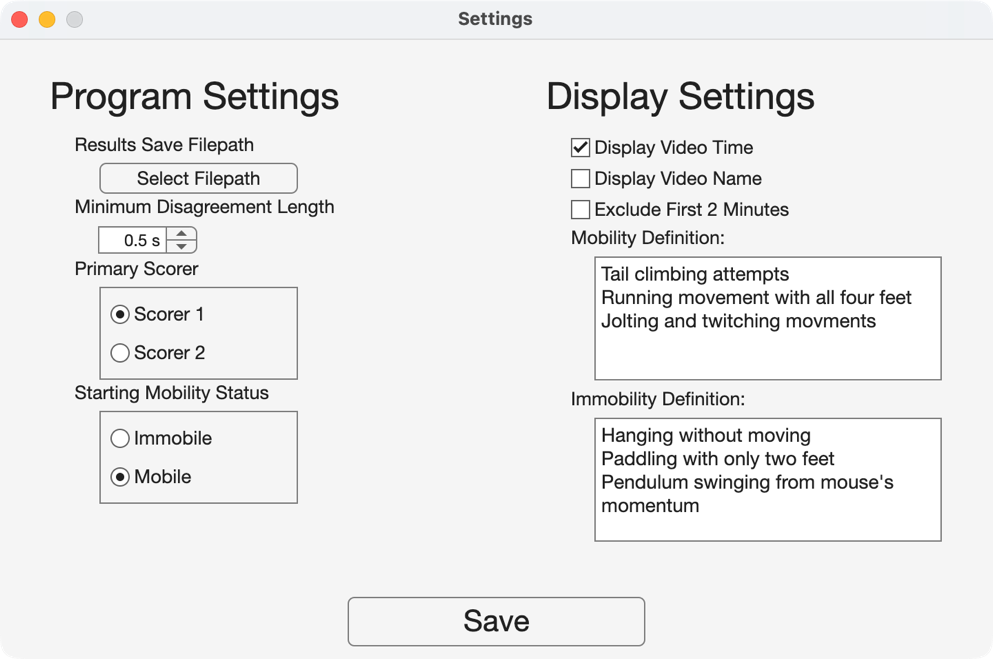


1. Program Settings:
   1. Click “Select Filepath” to set the file path where the results spreadsheet will be saved.
   2. ‘Minimum Disagreement Length’ and ‘Primary Scorer’ only apply to rescore mode.
   3. Choose the Starting Mobility Status, which sets the value (“Immobile” or “Mobile”) that the mobility switch will start on.
2. Display Settings:
   1. Display Video Time: If checked, the video time will be displayed while the video plays.
   2. Display Video Name: If checked, the video name will be displayed while the video plays.
   3. Exclude First 2 Minutes: If checked, the first 2 minute of the trial will be excluded.
   4. Mobility Definition: This text describes actions that should be deemed by the scorer as mobility. It will be displayed while the video plays.
   5. Immobility Definition: This text describes actions that should be deemed by the scorer as immobility. It will be displayed while the video plays.
3. Once all settings have been finalized, click “Save” to save settings.
4. Select “Score entire videos” and enter your name in the “Scorer Name” text box. Press “Next”.


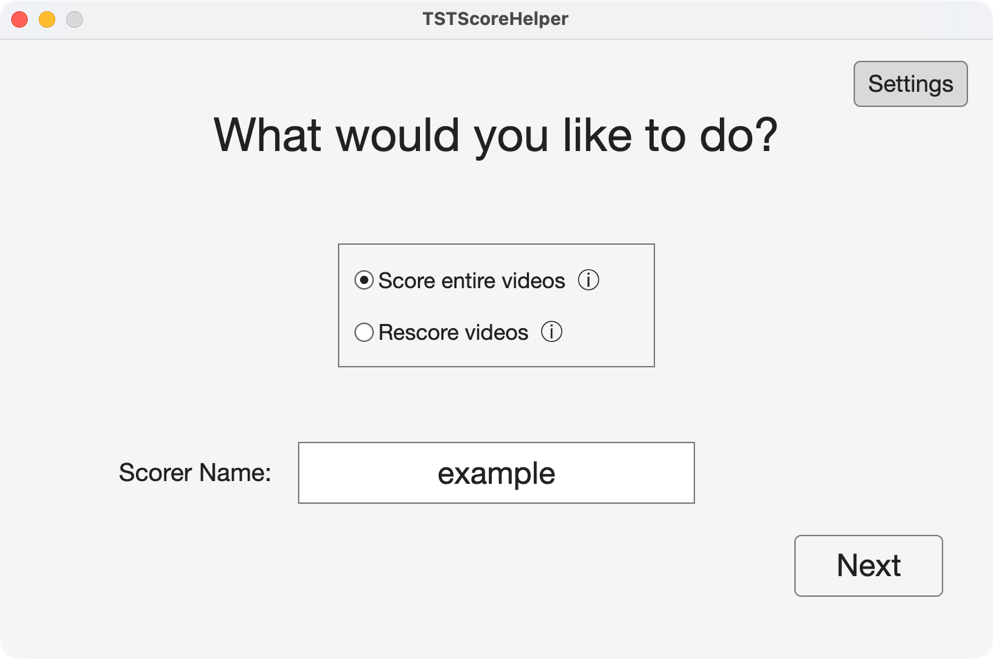


1. Press “Upload file” and select the scoring filepath spreadsheet. If scoring the included example videos, select the included example spreadsheet “example_scoring_filepath_spreadsheet”. **Note that the example spreadsheet needs to be modified to reflect the correct filepaths for your computer.**


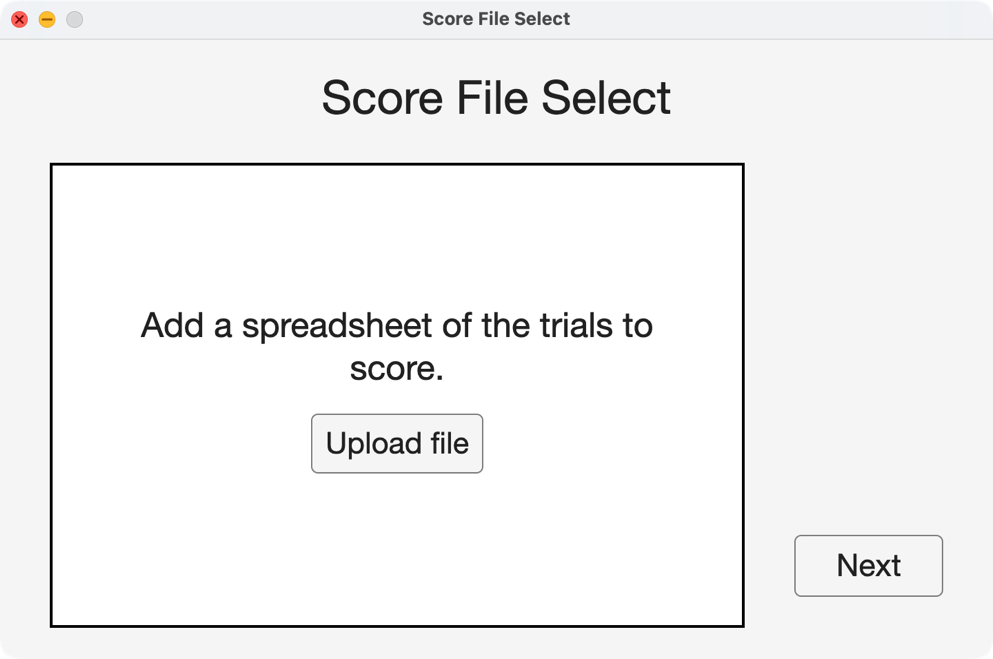


1. The list of video files that will be played will be displayed. Press “Next” to begin scoring.

*Note, the table cannot be edited with the GUI, so if it has an error, it must be removed, edited with a different application, and reuploaded. To remove the table, press “Delete table”.*


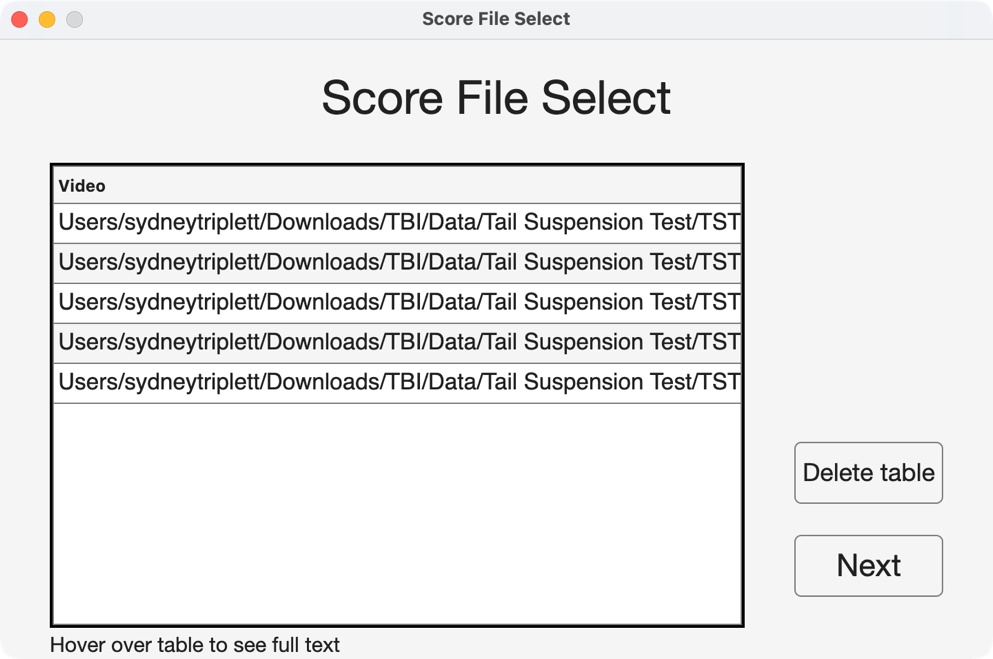


1. Press ”Start” to start scoring the first video.


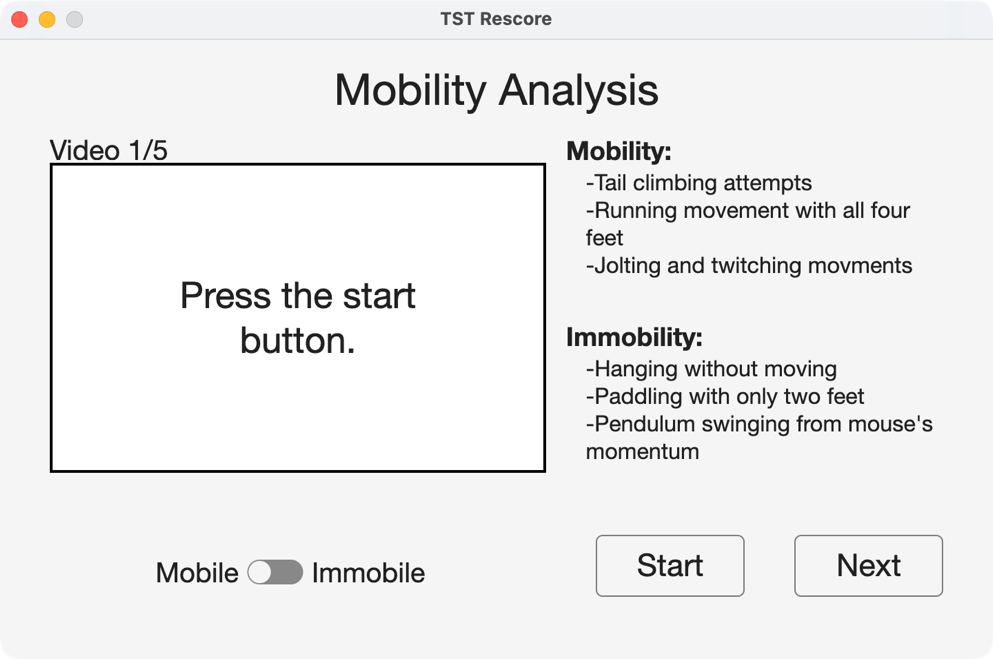


1. Toggle the “Mobile/Immobile” switch accordingly as the video plays.


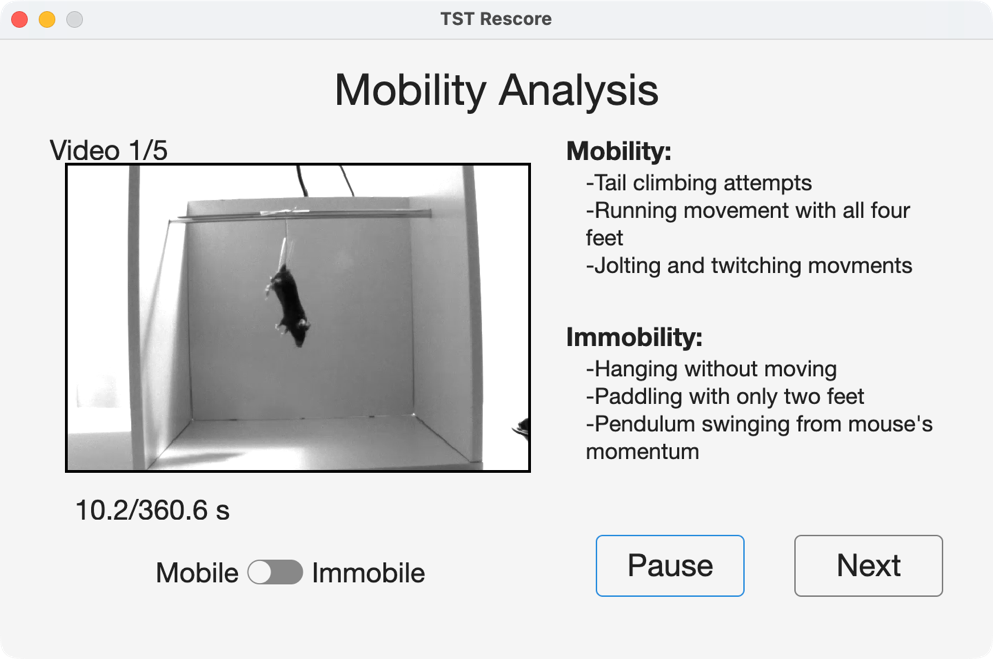


*Note, while the video plays, it can be paused by pressing the “Pause” button, and then un-paused by pressing the “Continue” button.*


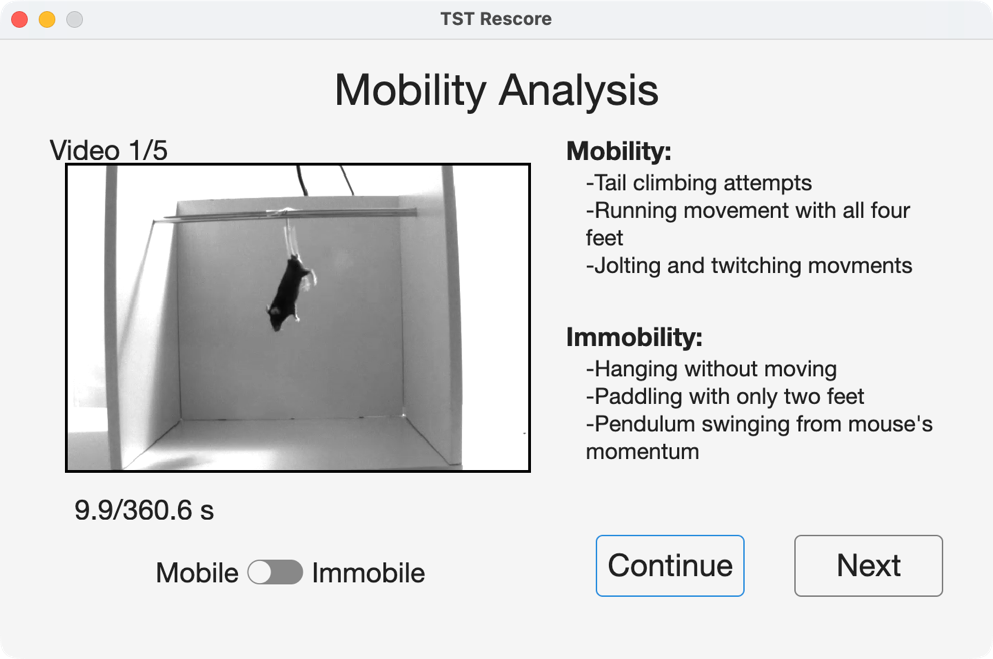


1. When the video ends, press “Next” to save the scoring timeline and queue up the next video.


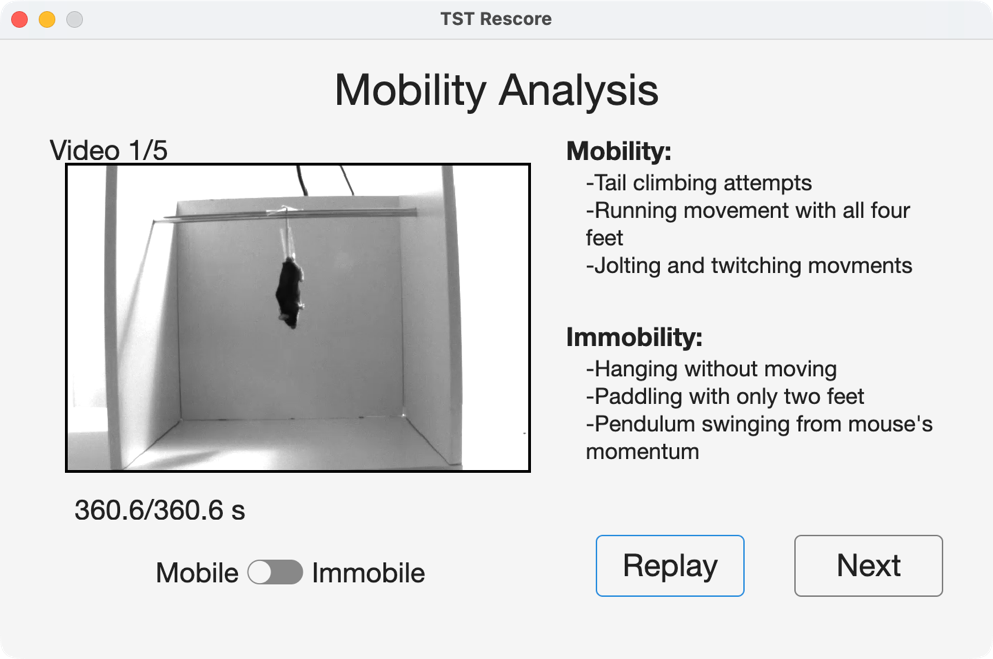


*Note, when the video ends, the “Pause” button text will change to “Replay”. If you for any reason want to rescore the video, click “Replay” to start the video over from the beginning and overwrite the scoring timeline just completed.*

1. After all videos are scored, a pop-up window will appear listing where the results spreadsheet is saved.


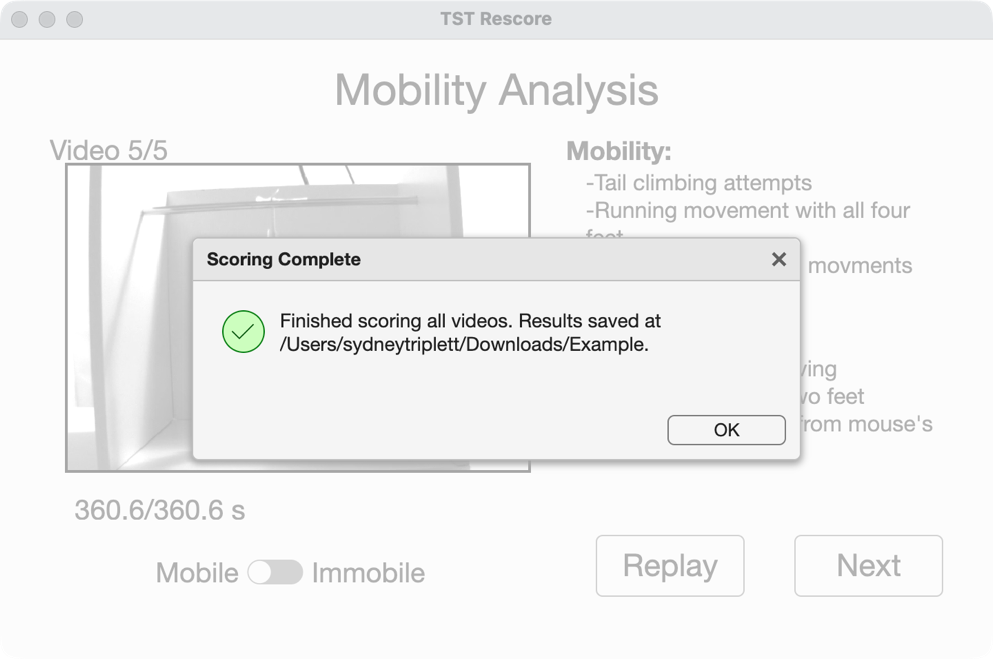


1. Close the pop-up window and the TST Score Helper GUI. Trying to open another instance of the GUI without closing the previous instance may cause errors.

# **TST Score Helper GUI in rescore mode: Example**

1. Open the software by double clicking on the file *TSTScoreHelper* (if running stand-alone software) or by running the TSTScoreHelper_FullCode.m script in the MATLAB command window (if running on MATLAB). The home page shown below should pop up.


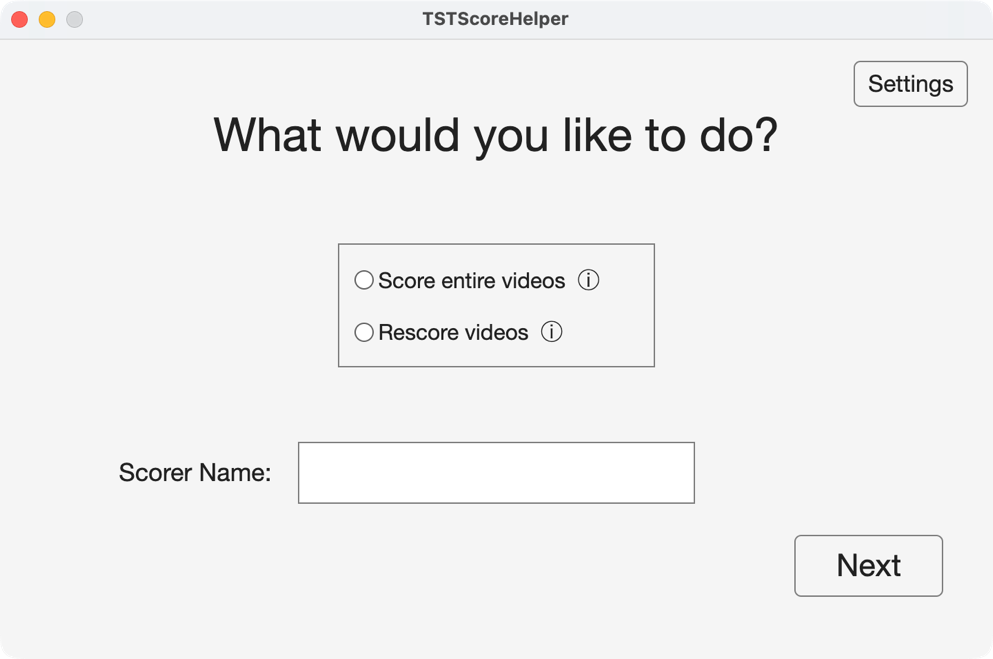


1. Press “Settings”. Settings should be adjusted every time the program is opened. The settings page with the default settings is shown below.


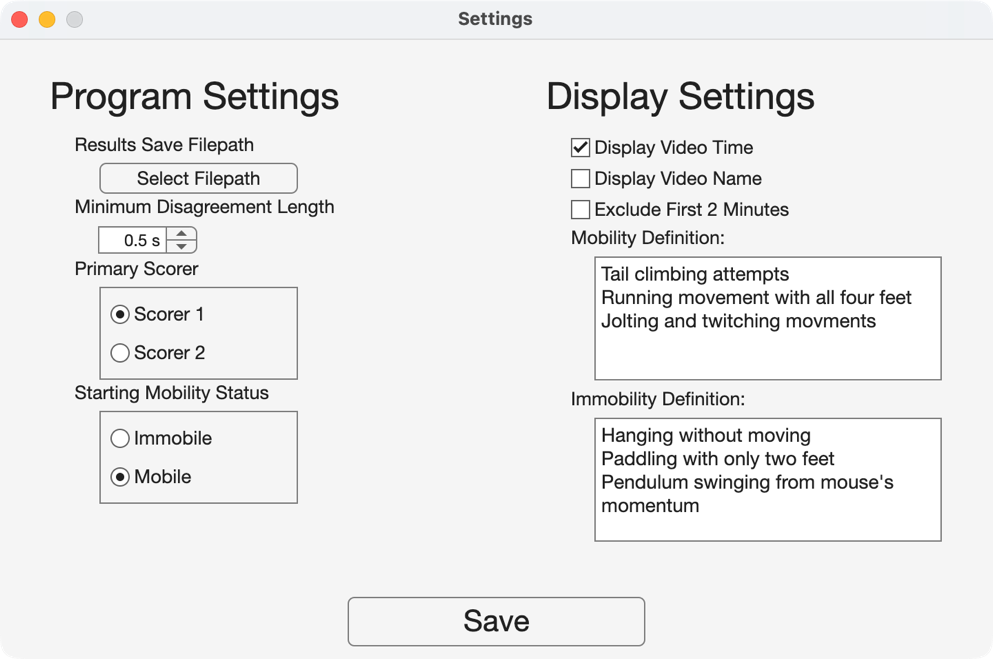


1. Program Settings:
   1. Click “Select Filepath” to set the file path where the results spreadsheet will be saved.
   2. Set the Minimum Disagreement Length, which sets the minimum duration (in seconds) that a period of disagreement must reach to be rescored.
   3. Choose the Primary Scorer as either Scorer 1 or Scorer 2, who’s scoring timeline will be used to fill in portions of trials not rescored.
   4. Choose the Starting Mobility Status, which sets the value (“Immobile” or “Mobile”) that the mobility switch will start on.
2. Display Settings:
   1. Display Video Time: If checked, the video time will be displayed while the video plays.
   2. Display Video Name: If checked, the video name will be displayed while the video plays.
   3. Exclude First 2 Minutes: If checked, the first 2 minute of the trial will be excluded.
   4. Mobility Definition: This text describes actions that should be deemed by the scorer as mobility. It will be displayed while the video plays.
   5. Immobility Definition: This text describes actions that should be deemed by the scorer as immobility. It will be displayed while the video plays.
3. Once all settings have been finalized, click “Save” to save settings.
4. Select “Rescore videos” and enter your name in the “Scorer Name” text box. Press “Next”.


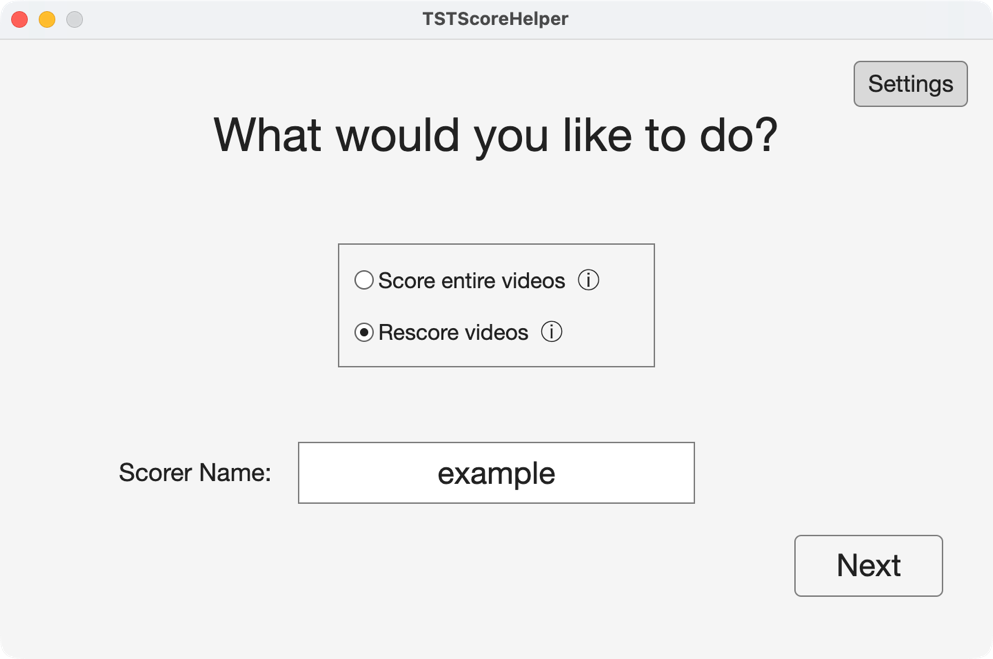


1. Press “Upload file” and select the rescoring filepath spreadsheet. If rescoring the included example videos, select the included example spreadsheet “example_rescoring_filepath_spreadsheet”. **Note that the example spreadsheet needs to be modified to reflect the correct filepaths on your computer.**


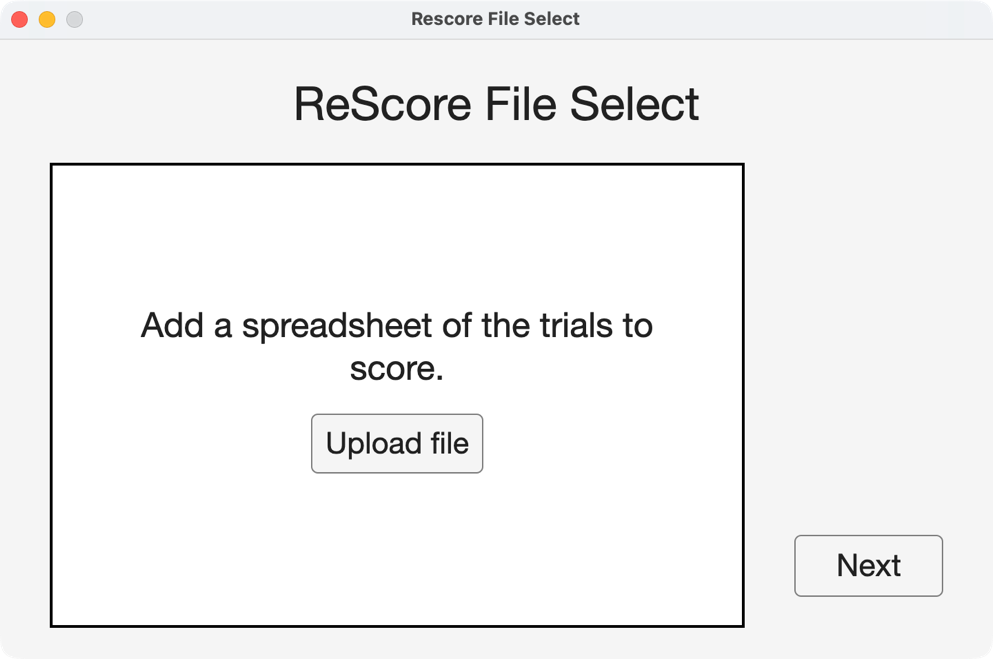


1. The list of video files that will be played will be displayed. Press “Next” to begin scoring.

*Note, the table cannot be edited with the GUI, so if it has an error, it must be removed, edited with a different application, and reuploaded. To remove the table, press “Delete table”.*


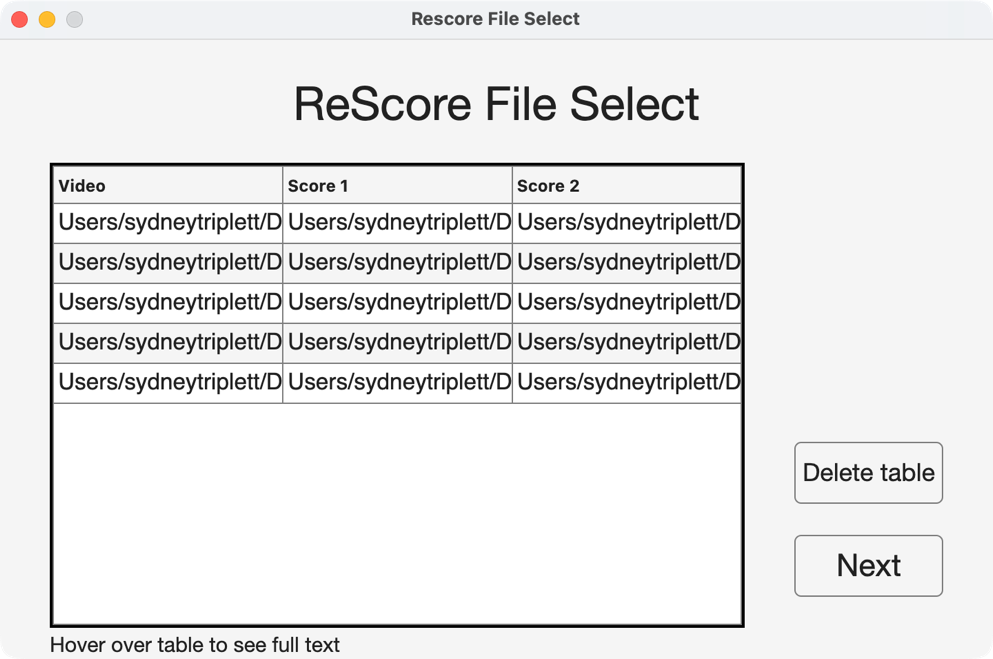


1. Press ”Start” to start the first clip of the first video.


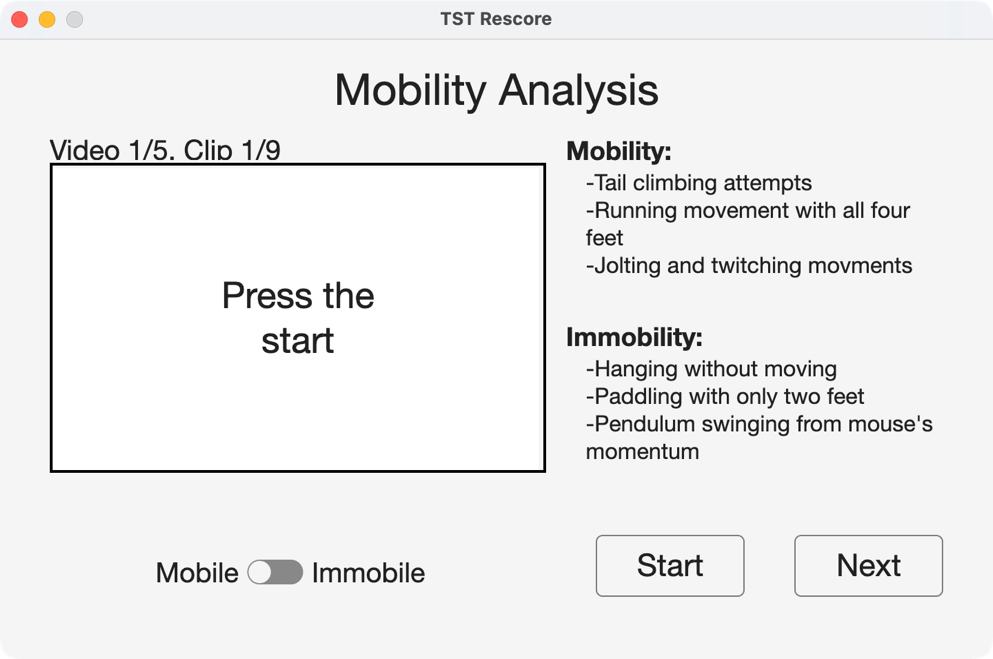


1. Watch the entirety of the clip and then select the appropriate mobility state with the “Mobile/Immobile” switch and press “Next” to confirm choice and queue up the next clip.


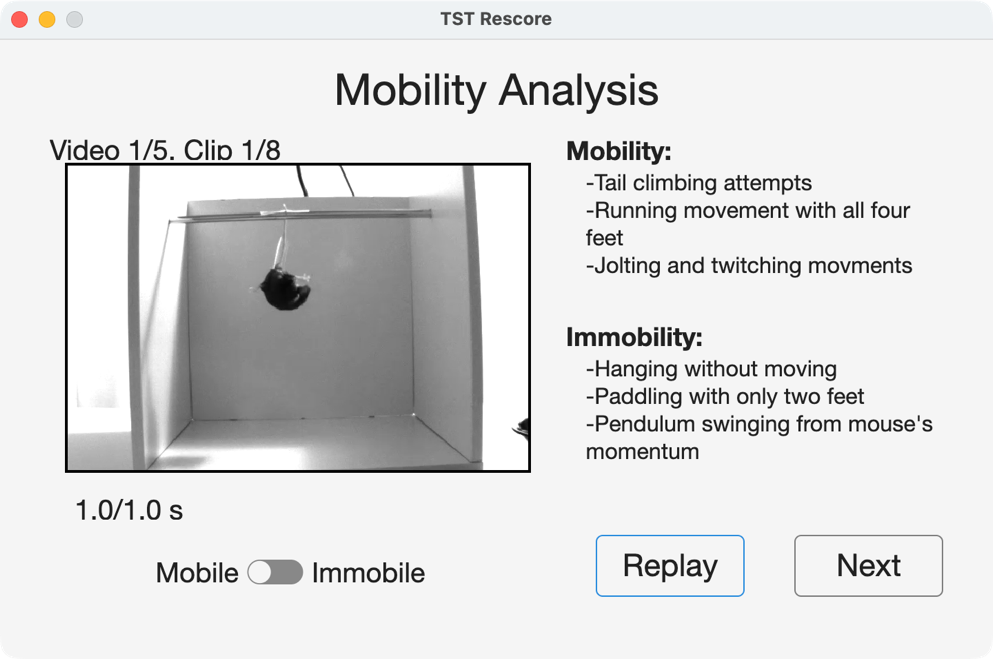


*Note, when the clip ends, the “Pause” button text will change to “Replay”. If you want to watch the clip again, click “Replay”.*

*Note, while the clip plays, it can be paused by pressing the “Pause” button, and then un-paused by pressing the “Continue” button.*


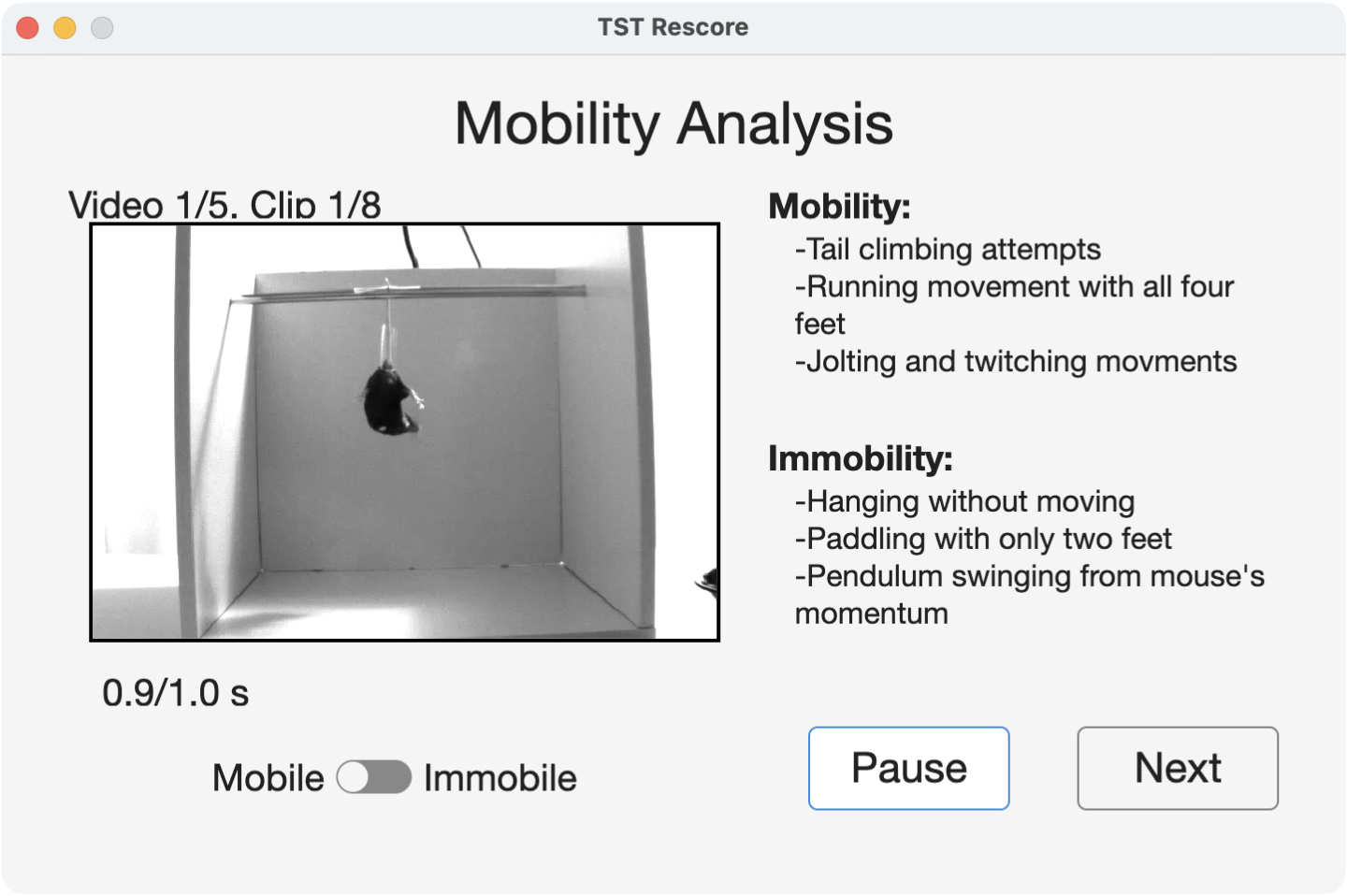


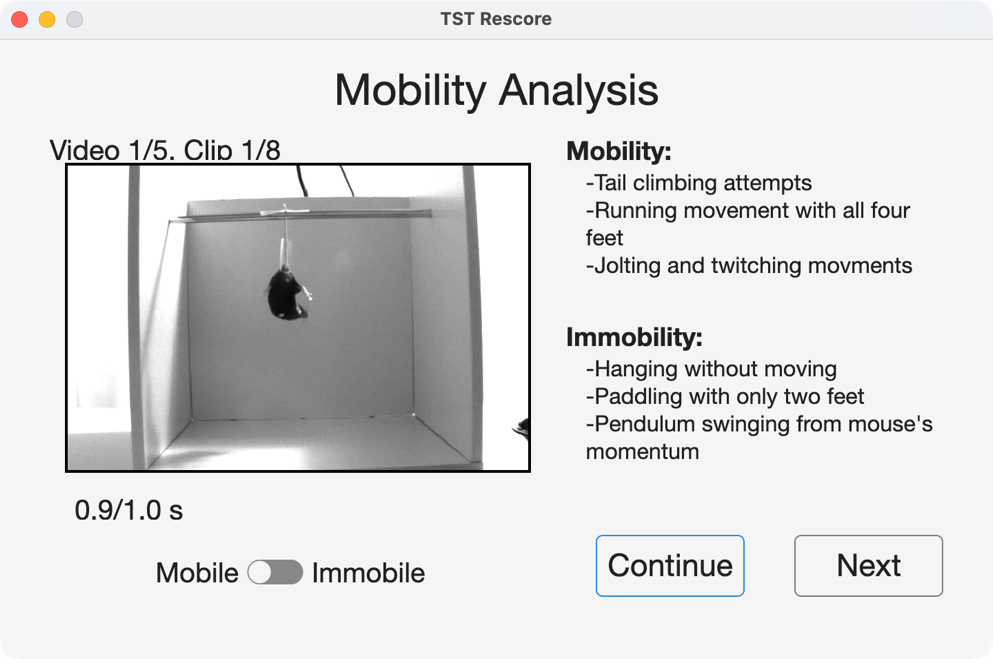


1. The GUI will iterate through all the clips in each video. After the final clip from the final video is scored, a pop-up window will appear listing where the results spreadsheet is saved.


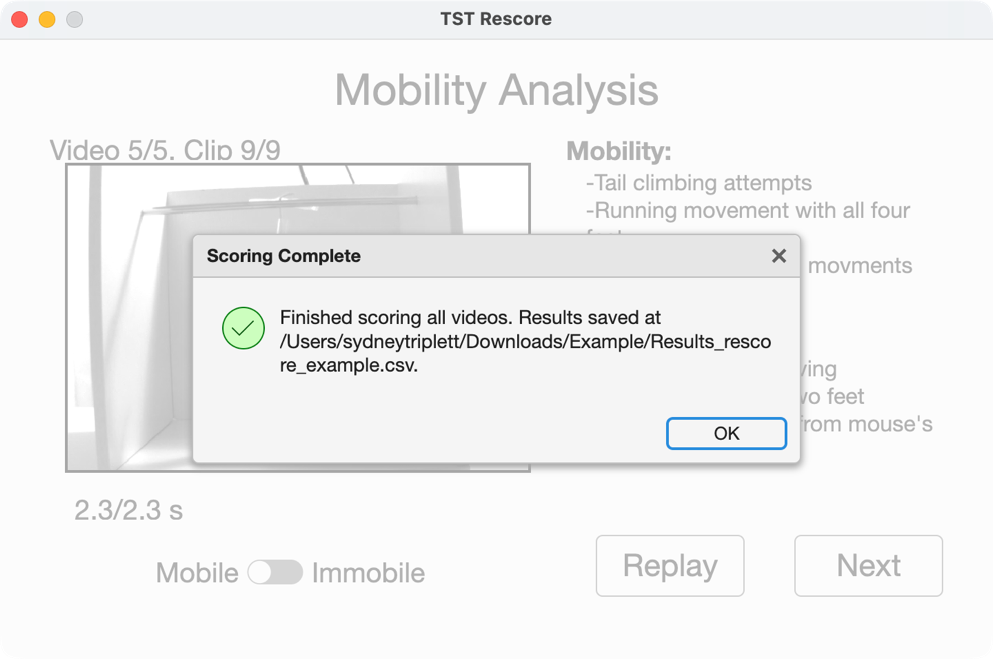


1. Close the pop-up window and the TST Score Helper GUI. Trying to open another instance of the GUI without closing the previous instance may cause errors.
